# Scaffold-Free Functional Deconvolution Identifies Clinically Relevant Metastatic Melanoma EV Biomarkers

**DOI:** 10.3390/cancers17152509

**Published:** 2025-07-30

**Authors:** Shin-La Shu, Shawna Benjamin-Davalos, Xue Wang, Eriko Katsuta, Megan Fitzgerald, Marina Koroleva, Cheryl L. Allen, Flora Qu, Gyorgy Paragh, Hans Minderman, Pawel Kalinski, Kazuaki Takabe, Marc S. Ernstoff

**Affiliations:** 1 Department of Medicine, Roswell Park Comprehensive Cancer Center, Buffalo, NY 14263, USA; 2 Frederick National Laboratory for Cancer Research, Frederick, MD 21701, USA; 3 New York Center of Excellence in Bioinformatics and Life Sciences, Buffalo, NY 14263, USA; 4 Department of Hepatobiliary and Pancreatic Surgery, Institute of Science Tokyo (Yushima Campus), Tokyo 13-8510, Japan; 5 Flow and Image Cytometry Resource, Roswell Park Comprehensive Cancer Center, Buffalo, NY 14263, USA; 6 Department of Surgical Oncology, Roswell Park Comprehensive Cancer Center, Buffalo, NY 14263, USA

**Keywords:** melanoma, metastasis, tumor microenvironment (TME), extracellular vesicles (EV), proteomics, REIUS isolation, disease-free survival (DFS), overall survival (OS), biomarker discovery

## Abstract

**Background:** Melanoma metastasis, driven by tumor microenvironment (TME)-mediated crosstalk facilitated by extracellular vesicles (EVs), remains a major therapeutic challenge. A critical barrier to clinical translation is the overlap in protein cargo between tumor-derived and healthy cell EVs. **Objective:** To address this, we developed Scaffold-free Functional Deconvolution (SFD), a novel computational approach that leverages a comprehensive healthy cell EV protein database to deconvolute non-oncogenic background signals. **Methods:** Beginning with 1915 proteins (identified by MS/MS analysis on an Orbitrap Fusion Lumos Mass Spectrometer using the IonStar workflow) from melanoma EVs isolated using REIUS, SFD applies four sequential filters: exclusion of normal melanocyte EV proteins, prioritization of metastasis-linked entries (HCMDB), refinement via melanocyte-specific databases, and validation against TCGA survival data. **Results:** This workflow identified 21 high-confidence targets implicated in metabolic-associated acidification, immune modulation, and oncogenesis, and were analyzed for reduced disease-free and overall survival. SFD’s versatility was further demonstrated by surfaceome profiling, confirming enrichment of H7-B3 (CD276), ICAM1, and MIC-1 (GDF-15) in metastatic melanoma EV via Western blot and flow cytometry. Meta-analysis using Vesiclepedia and STRING categorized these targets into metabolic, immune, and oncogenic drivers, revealing a dense interaction network. **Conclusions:** Our results highlight SFD as a powerful tool for identifying clinically relevant biomarkers and therapeutic targets within melanoma EVs, with potential applications in drug development and personalized medicine.

## Simple Summary

Melanoma is a serious skin cancer that can spread to other parts of the body. This research focused on tiny particles called extracellular vesicles (EVs) released by melanoma cells. By carefully analyzing these EVs, the study identified specific proteins that are much more common in melanoma EVs compared to EVs released by normal cells. These proteins, including ICAM-1, SPPL2A, CD276, and CD44, may help the cancer cells stick together, spread, and avoid the immune system. The findings suggest that these EV proteins could be used to help doctors predict how aggressive a melanoma is and potentially guide new treatment approaches, opening the door to more targeted therapies that could improve outcomes for patients with advanced melanoma.

## 1. Introduction

Melanoma metastasis is a hallmark of exceptional biological aggression even among cancers [1]. Melanoma metastasis is a multifaceted process that requires dynamic interactions between cancer cells and their microenvironment. Metastasis involves the generation of a pre-metastatic niche, followed by invasion, entry, and survival in the blood vessels, and then attachment to and colonization of the host organs [2]. Lymphatic flow and chemotaxis are believed to be the main homing beacons for metastasis of melanoma to distal sites from the primary tumor [3]. The initial phases of microenvironmental reprogramming (acidification, immune evasion, and stromal remodeling) not only drive local tumor progression but also establish a pre-metastatic niche in distant tissues, and are a process mediated by virus-sized, nanoscale couriers collectively known as extracellular vesicles (EV). Within a subset of EV, small vesicles of endosomal origin secreted by tumors are known as tumor-derived exosomes (TEX). TEX culture the tumor microenvironment (TME) through long-distance signaling (via peripheral blood and lymphatic systems) to suppress the immune response by local stromal remodeling and acidification. TEX have become targets for investigation, understanding, and intervention aimed at impairing metastasis [4,5,6,7,8]. Emerging evidence suggests that TEX may serve as an “engineer brigade” ferrying large quantities of function proteins such as metabolically acidifying enzymes, immune checkpoint proteins, and stromal-modifying factors to remotely precondition the distal sites for colonization. Like a convoy of constant supply, TEX can provide the critical quantities of material required for large-scale, sustained yet peripheral engineering tasks such as weakening structural barriers through acidification [9], sustaining supply lines through lipid provisioning [10], and camouflaging sites through immune checkpoint deployment [11].

Quantifying exosomal proteomes remains challenging, as traditional normalization strategies (e.g., total protein or cell count) may confirm protein presence but often fail to account for biological relevance or functional heterogeneity [12]. Exosomes encapsulate a sub-stoichiometric subset of their cellular proteome, likely reflecting active sorting mechanisms and non-canonical cargo acquisition [13,14]. Previous approaches focused on comparative analysis between different exosome populations, such as distinguishing small versus large vesicles [15] or identifying tissue-specific markers by comparing exosomes from different cancer cell lines [16]. Subsequent methodological advances introduced deconvolution techniques to determine cellular origins of circulating exosomes and remove technical contaminants such as co-isolated plasma proteins [17]. Other studies have employed direct disease-versus-control comparisons, contrasting exosomal protein profiles between disease and healthy cohorts to identify differentially expressed proteins (DEPs) [18].

However, such approaches neither prioritize the identification of proteins uniquely present in tumor-derived exosomes (i.e., regardless of abundance thresholds) nor evaluate the association between exosomal proteome composition and clinically relevant endpoints of metastatic progression, such as overall survival (OS) and disease-free survival (DFS). Low-abundance or non-quantitative markers may serve as robust indicators of pathological states when absent from the circulating exosomes of healthy individuals [19]. This paradigm shift offers the potential to uncover actionable candidates whose mere presence in the extracellular milieu signals oncogenic transformation or metastatic dissemination.

Using a well-established, commonly employed method in infectious pathology proteomics but not in DEP-dependent cancer proteomics, we identified several biochemically validated biomarker targets on EVs. These targets were detectable by Western blot, and in the case of CD276, also by flow cytometry.

## 2. Materials and Methods

### 2.1. Cell Culture

Human neonatal melanocytes from four different donors (HEMn, ATCC, Manassas, VA, USA) were cultured in Dermal Cell Basal Medium (ATCC) supplemented with a Melanocyte Growth Kit (ATCC), 1 mM Corning glutaGRO, and 1% antibiotic solution (100 U/mL penicillin, 100 mg/L streptomycin, Corning Life Sciences, Tewksbury, MA, USA). Melanoma cell lines 2183-Her4 (BRAF^WT^), 888-mel, and 526-mel (both BRAF^V600E^ mutants) were obtained from the National Institutes of Health (NIH) and cultured in three 15 cm Corning plates using RPMI 1640 medium supplemented with 5% exosome-depleted FBS (Gibco/ThermoFisher Scientific, Waltham, MA, USA), Glutamax (Gibco/ThermoFisher Scientific), and Penicillin/Streptomycin (Gibco/ThermoFisher Scientific) at 37 °C/5% CO_2_. When approximately 70% confluent, cell monolayers were washed with plain RPMI 1640 and incubated overnight in 15 mL RPMI without FBS before supernatant harvest for exosome isolation.

### 2.2. Exosome Isolation

Supernatants from the three plates of melanocytes or 888-mel were collected and pooled, and exosomes were isolated using the REIUS method [20]. Briefly, supernatants were centrifuged at 300× *g* for five minutes to pellet cells, followed by centrifugation at 3000× *g* to pellet cell debris. The supernatants were passed through a 0.20 µm PES syringe filter and concentrated using a 100 kD MWCO Ultracel-100 Amicon^®^ Ultra-15 Centrifugal Filter Unit (or 3 kD MWCO Ultracel-3 for “3 kD + SEC” exosomes, Millipore Sigma, Burlington, MA, USA) at 3000× *g* to concentrate the 45 mL down to approximately 100 µL. The resulting concentrate (termed CONC when Ultracel-100 filters were used) was then subjected to SEC using Exo-spin™ columns (Cell Guidance Systems, Cambridge, UK) according to the manufacturer’s instructions. Exosomes were collected into Corning™ Costar™ Low Binding Microcentrifuge tubes. The 100 kD UF flow-through (FT) was also collected but required concentration using 3 kD MWCO ultrafilters to reduce sample volume. Exosome counts were determined by performing nanoparticle tracking analysis using ZetaView (Particle Metrix GmbH, Bavaria, Germany), and intact vesicular morphology was confirmed using Transmission Electron Microscopy (TEM) as described previously [21,22].

### 2.3. Sample Preparation for LC-MS Analysis

Lysis buffer (50 mM Tris-FA, 150 mM NaCl, 0.5% sodium deoxycholate and 2% SDS, 2% NP-40, pH 8.0) containing cOmpleteTM protease inhibitor and PhosSTOP phosphatase inhibitor (Roche Applied Science, Indianapolis, IN, USA) was added to exosome isolates, centrifuged at 20,000× *g* for 30 min at 4 °C and the resulting protein containing supernatants were stored at −80 °C until analysis. The protein extracts were processed using a Surfactant Cocktail-Aided Extraction/Precipitation/On-Pellet Digestion (SEPOD) [23], which provides extensive cleanup to remove detergents and non-protein matrix components, as well as deep protein denaturation (by both surfactants and precipitation) for rapid, efficient, and reproducible digestion, thereby achieving reliable quantification across all samples.

A 50 μL aliquot containing 100 μg of extracted protein was diluted with an equal volume of 50 mM Tris-formic acid (FA) buffer (pH 8.5). Then, 200 mM DTT was added to a final concentration of 10 mM. After incubation at 56 °C for 30 min in an Eppendorf Thermomixer (Eppendorf SE, Hamburg, Germany), 500 mM Iodoacetamide (IAM) solution was added to each sample to a final concentration of 20 mM. Samples were then incubated at 37 °C for 30 min in darkness with rigorous oscillation. For pellet precipitation, one volume of chilled acetone (−20 °C) was gently added to each sample and mixed for 1 min to obtain a cloudy suspension. Then, 8 volumes of chilled acetone were added to the mixture to precipitate proteins. The solution was vortexed until it became clear and was stored at −20 °C overnight to allow complete precipitation. Subsequently, samples were centrifuged at 20,000× *g* for 30 min at 4 °C to obtain a protein pellet. After removing the supernatant, 500 μL of methanol was added to wash the pellet. The tube was manipulated to enable the methanol to cover the pellet. Samples were centrifuged for 3–5 min; the supernatant was discarded, and the protein pellet was allowed to air dry.

For protein digestion, the pellet was dissolved in 80 μL Tris-FA (pH 8.5) buffer and sonicated in a water bath at 37 °C. Then, 20 μL Tris-FA buffer containing activated trypsin (SigmaAldrich/MilliporeSigma, Saint Louis, MO, USA) was added to the samples at a ratio of 1:40 (enzyme:substrate) and incubated overnight at 37 °C in an Eppendorf Thermomixer. To terminate the digestion, 1% formic acid was added and gently vortexed, followed by centrifugation at 20,000× *g* for 20 min at 4 °C and careful transfer of 2/3 of the digestion solution to a vial for LC-MS analysis.

### 2.4. Nano LC and High-Field Orbitrap MS

The nano-RPLC system, consisting of a Dionex Ultimate 3000 nano-LC and an UltiMate 3000 Gradient Micro-LC System with a WPS-3000 autosampler, was employed. Peptide separation employed a long nano-LC column (75 μm ID × 100 cm) with Pepmap 3 μm C18 particles. A large-ID trap (300 μm ID × 1 cm) was packed with Zorbax 5 μm C18 materials to allow large-capacity loading and removal of hydrophobic and hydrophilic matrix components. Mobile phase A was 0.1% formic acid in 2% acetonitrile, and mobile phase B was 0.1% formic acid in 88% acetonitrile. A 4 μg peptide sample was loaded onto the trap with 1% B at 10 μL/min. After the trap was washed for 3 min, a 250 nL/min flow rate was used to back-flush the samples onto the nano-LC column for further separation. The column was enclosed in a heating sheath filled with heat-conductive silicone and warmed homogeneously at 52 °C, which helps improve the chromatographic resolution and reproducibility. The following was the 2.5 h separation gradient used on the column: 4% B for 15 min; 13–28% B for 110 min; 28–44% B for 5 min; 44–60% B for 5 min; 60–97% B for 1 min; 97% B for 17 min.

An Orbitrap Fusion Lumos Mass Spectrometer (ThermoFisher Scientific) was employed for peptide identification and quantification. Data collection was operated in a 3 s cycle with a data-dependent top-speed mode. MS1 survey scan (*m*/*z* 400–1500) was at a resolution of 120,000, with automated gain control (AGC) target of 500,000 and a maximum injection time of 50 ms. Precursors were fragmentized in HCD activation mode at a normalized collision energy of 35% and the dynamic exclusion was set with 45 s. Precursors were filtered by quadrupole using an isolation window of 1 Th. The MS2 spectra were collected at a resolution of 15,000 in the Orbitrap, with an AGC target of 50,000 and a maximum injection time of 50 ms.

### 2.5. Protein Identification and Quantification

The MS-GF+ searching engine was employed to identify peptides by scoring MS/MS spectra against peptides derived from the UniProt-SwissProt protein database (Homo sapiens, 20,212 entries, released in July 2015). A total of 4061 proteins was identified. The search parameters were set to 20 ppm tolerance for precursor ion mass and 0.02 Da for fragment ion mass. Two missed cleavages were permitted for fully tryptic peptides. Carbamidomethylation of cysteine was set as a static modification, and a dynamic modification was defined as oxidation at methionine and acetylation at the N-terminal. The FDR of identification was estimated using a target-decoy search strategy that was dependent on a concatenated database of forward and reverse sequences. At least two distinct peptides were required for each identified protein. The FDR for peptide and protein identification was set to 0.1% and 1%, respectively.

Quantitative analysis was performed with the locally developed IonStar pipeline [24]. It incorporates SIEVE (v2.2, Thermo Scientific) for chromatographic alignment using the ChromAlign algorithm, extraction and procurement of peptide peak areas, and a locally developed R package version 3.4.4 (available at https://github.com/shxm725/IonStarstat—accessed on 27 July 2025) for data quality control, aggregation, normalization, removing outliers, and summarization. The ion-current area under the curve (AUC) of same peptide was integrated into the same frame according to the following criteria: *m*/*z* width = 10 ppm; frame time width = 2.5 min. The merged feature intensity file was further processed using the IonStar data processing pipeline for proper data normalization (i.e., total ion-current normalization) and summarization (i.e., sum of intensities). Peptides shared by different proteins were excluded in order to quantify only the unique peptides of each protein. The relative expression ratios of proteins in two groups were calculated by comparing average ion current intensities of replicates in each group, and the statistical significance was evaluated with ANOVA. The cut-off for altered proteins and false positive altered protein discovery rate were established using an Experimental Null method [25].

### 2.6. Functional Analysis

Gene Ontology (GO) annotation and KEGG pathway enrichment analysis were performed using DAVID Bioinformatics Resources v6.7 (https://david.ncifcrf.gov/—accessed on 27 July 2025). Enrichment for specific GO terms and KEGG pathways was identified using DAVID’s modified Fisher’s exact test (EASE score), and *p*-values were corrected for multiple testing using the Benjamini–Hochberg method. GO terms and pathways with a false discovery rate (FDR) adjusted *p*-value < 0.05 were considered statistically significant. All reported categories met this threshold.

### 2.7. Exosome Capture Using Magnetic Beads

To visualize exosome surface protein expression, exosomes derived from 888-mel were linked to streptavidin-coated magnetic beads (ExoCap Streptavidin Kit, MBL International, Woburn, MA, USA), and microcentrifuge tubes and pipette tips used throughout were low-binding. Briefly, the beads (approx. 1 × 10^7^ per assay) were washed twice using wash buffer (MBL) by magnetic capture and then incubated with rotation overnight at 4 °C with 1 µg of biotinylated antibody. Biotin anti-human CSPG-4 antibodies (Dr. Soldarno Ferrone’s lab, Department of Surgery at Massachusetts General Hospital / Harvard Medical School, Boston, MA, USA) and CD276 (BioLegend, San Diego, CA, USA) were used to create an exosome capture cocktail, and a biotin-IgG1 isotype (Abcam, Cambridge, UK) was used as a control. The next day, the beads were washed twice with wash buffer and then resuspended in treatment buffer (MBL). A titrated concentration of 888-mel-derived exosomes was added to the beads, and the samples were incubated for 18–24 h with rotation at 4 °C to allow for exosome binding. Finally, the samples were washed three times by magnetic capture to remove any unbound exosomes and were resuspended in 250 µL PBS.

### 2.8. Western Blot Analysis of Melanoma-Specific Surface Proteins

**Figure 1:**
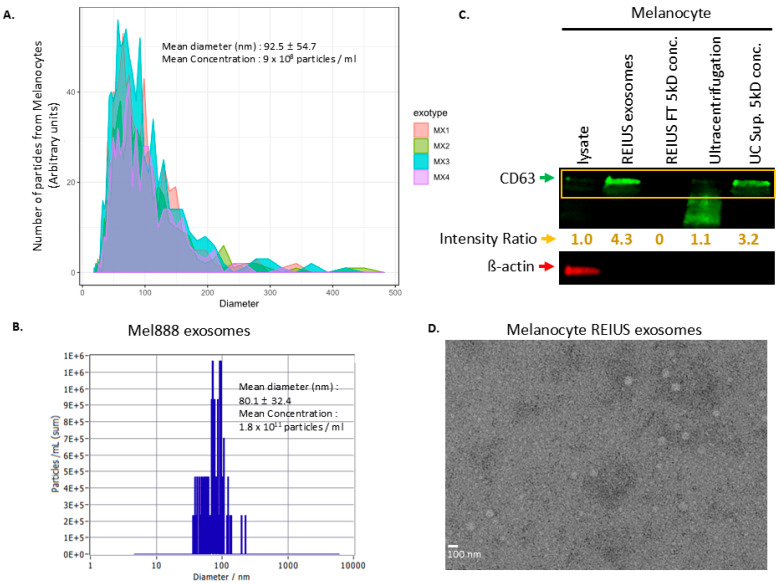
Comprehensive characterization of extracellular vesicles (EVs) isolated from normal melanocytes cells using the REIUS method. (**A**) Nanoparticle tracking analysis (NTA) profiles of EVs from four melanocyte exotypes (MX1–MX4), showing particle size distribution and concentration (mean diameter: 92.5 ± 54.7 nm; mean concentration: 9 × 10^8^ particles/mL). (**B**) Nano-Tracking Analysis (NTA) profile of Mel888 melanoma-isolated REIUS exosomes, analyzed at the same time with melanocytes as biological reference. (**C**) Western blot analysis of melanocyte-derived fractions for EV marker CD63 and loading control β-actin, comparing lysate, REIUS EV, REIUS FT 5 kD conc. (FT = flowthrough, i.e., REIUS isolation concentrated using 5 kD MWCO filtration spin columns to check for isolation failure) ultracentrifugation (UC) pellet, and UC Sup. 5 kD conc (UC supernatant concentrated using 5 kD MWCO filtration spin columns to check for isolation failure). (**D**) Transmission electron microscopy (TEM) image of melanocyte REIUS EVs, confirming vesicular morphology and size (scale bar: 100 nm).

Cells were lysed with RIPA Lysis and Extraction Buffer containing 1X Halt™ Protease and Phosphatase Inhibitor Cocktail (ThermoFisher Scientific) according to manufacturer’s instructions. Exosomes were diluted 1:5 in RIPA containing Halt protease and phosphatase inhibitors, and protein concentration of both the cell lysates and exosomes was determined using the Pierce™ BCA Protein Assay Kit (ThermoFisher). Protein (20 µg) was electrophoresed by SDS-PAGE using 4–20% Mini-PROTEAN^®^ TGX™ Gels (Bio-Rad Laboratories, Hercules, CA, USA) and transferred onto low fluorescence PVDF (EMD Millipore, Burlington, MA, USA) using a Trans-Blot Turbo Transfer System (Bio-Rad). The membrane was blocked for one hour at room temperature (RT) using AdvanBlock-Chemi (Advansta, San Jose, CA, USA) and then incubated overnight with primary antibody: anti-CD63 (Sigma-Aldrich), anti- β-actin (Cell Signaling Technology, Danvers, MA, USA), anti-IGFBP3 (R&D Systems, Minneapolis, MN, USA), anti-NCAPG (Abcam), anti-SPPL2A (Abcam), anti-SLC19A1 (MilliporeSigma), anti-TRAF3 (Cell Signaling), anti-TTPA (Abcam), anti-MERTK (Thermo Fisher Scientific, Waltham, MA, USA), anti-CD39 (Thermo Fisher Scientific), anti-MRP2 (Cell Signaling), anti-CD26 (Cell Signaling), anti-SMPD-1 (R&D Systems), anti-CD276 (Biolegend), anti-MIC-1 (R&D Systems), or anti-ICAM1. Following washes in 1X TBS containing 0.1% Tween, the membrane was incubated for 1 h at RT in AdvanBlock-Chemi buffer containing HRP-conjugated secondary antibody at a 1:1000 dilution: anti-mouse IgG (Invitrogen, Carlsbad, CA, USA), anti-Goat IgG (Invitrogen), or anti-Rabbit IgG (Invitrogen). The membrane was washed in TBS/Tween, and protein expression was visualized using WesternBright Sirius HRP substrate (Advansta) and the ChemiDoc™ Touch Imaging System and Image Lab Software version 3.0 (Bio-Rad). For Figure 1C, LI-COR compatible antibodies IRDye 680RD and 800CW (LI-COR Biosciences, Lincoln, NE, USA) for dual-florescence imaging were used instead of HRP-based fluorescence method.

### 2.9. Flow Cytometry

To assess the surface expression of melanoma proteins on exosomes, the captured exosomes were analyzed by flow cytometry. Approximately 0.4 µg of exosomes was used per analysis, whereby 20 µL of the 250 µL resuspended sample was used for each detection antibody. Samples were brought up to 100 µL in PBS and blocked for 10 min at RT with Human TruStain FcX™, an Fc receptor blocking solution (BioLegend). The samples were then labeled with the desired fluorochrome-conjugated detection antibodies in individual tubes and incubated in the dark for 1 h with rotation. The samples were washed twice with PBS, resuspended in 250 µL PBS, and transferred to 5 mL polystyrene tubes for analysis on a LSRFortessa™ Flow Cytometer FACSDiva version 8.0.2 (BD Biosciences, Milpitas, CA, USA) and FCS Express version 6 (De Novo Software, Pasadena, CA, USA).

### 2.10. Survival Analysis

TCGA melanoma cohort was downloaded through cBioportal [26,27]. Among 472 melanoma patients with gene expression data from RNA-seq, there are 103 patients with primary tumors. All 103 patients have overall survival, and 89 patients have disease-free survival information. Overall survival was defined as the time from surgery to any cause of death, and disease-free survival was defined as the time from surgery to recurrence of tumor or any cause of death. The median follow-up time was 14.6 months. Survival analyses of each gene were performed gene expression continuous value using Cox regression model. Proportionality assumption was confirmed using Schoenfeld residuals.

### 2.11. Statistical Analysis

The *t* test was used to determine whether the molecular weight of proteins is statistically different between REIUS and 3 kD + SEC. Boxplot was obtained using the ggplot2 package. All statistical analyses were conducted using R software version 3.4.4 (http:///www.r-project.org/—accessed on 27 July 2025) and Bioconductor version 3.12 (http://bioconductor.org/—accessed on 27 July 2025).

### 2.12. Network Analysis

STRING database tools version 12.5 (https://string-db.org—accessed on 27 July 2025) were used to map interactions among metastatic exosome proteins, highlighting functional clusters linked to metabolic reprogramming, immune modulation, and oncogenic signaling.

## 3. Results

For technical clarity on definition, this study defines exosomes as a subtype of EV that is gently isolated by REIUS method with negligible contaminating, non-exosomal soluble factors [22], but given that on the prospects of this study is to apply a proteomic pipeline to broad categories of EV databases found online, the terms “exosomes” will be reserved for experimental results derived from REIUS and “EV” will refer to vesicles obtained from non-REIUS methods that are used both in the study and in the literature for meta-analysis.

### 3.1. Metastatic Proteins from REIUS-Derived Exosomes

Isolating melanocyte exosomes is challenging, as the quantity of exosomes released is relatively sparse. Metastatic melanoma cells exhibit enhanced exosome shedding activity compared to normal melanocytes, with melanoma exosomes releasing vesicles more efficiently and with denser protein payload than melanocyte counterparts, where the same number of cells generates almost 2-log difference in exosome count in supernatant, and this is observed both in the literature and in our isolation (Figure 1A vs. Figure 1D) [28,29]. Using conventional ultracentrifugation (UC) methods, which are known to have limiting yields, can be particularly challenging for isolating melanocyte exosomes. Despite the similar mean diameters of exosomes derived from melanocytes and melanoma cells (melanocyte exosome mean diameter: 92.5 nm vs. Mel888 exosome mean diameter: 80.1 nm), it appears that melanocyte-derived extracellular vesicles (EVs) are structurally fragile when subjected to high centrifugal forces. This is evidenced by the “smear” of CD63 with variable molecular weight observed in the ultracentrifugation lane (Figure 1C; uncropped Western blots are shown in Figure S2). To further investigate, the UC supernatant was concentrated using a 5 kD MWCO filtration column, which captures both EVs and proteins larger than 5 kD. Notably, CD63 protein was still detectable in the UC supernatant, and it is unclear if the vesicles are structurally intact. In contrast, the REIUS flow-through when similarly concentrated showed no detectable CD63 expression, indicating that the REIUS method is more effective at isolating intact melanocyte EVs. The β-actin loading control was only present in the lysate, confirming the specificity of the exosome isolation. Collectively, these findings suggest that ultracentrifugation is not suitable for isolating melanocyte-derived EVs, likely due to their fragility, while the REIUS method, which utilizes a gentle isolation process (maximum 2000× *g*), enables effective recovery of intact exosomes. When examined under a TEM, these REIUS-isolated melanocyte exosomes exhibit well-preserved, distinct vesicular morphologies as shown in Figure 1D.

### 3.2. Classification of Proteomic Data Derived from REIUS Isolated Exosomes

**Figure 2:**
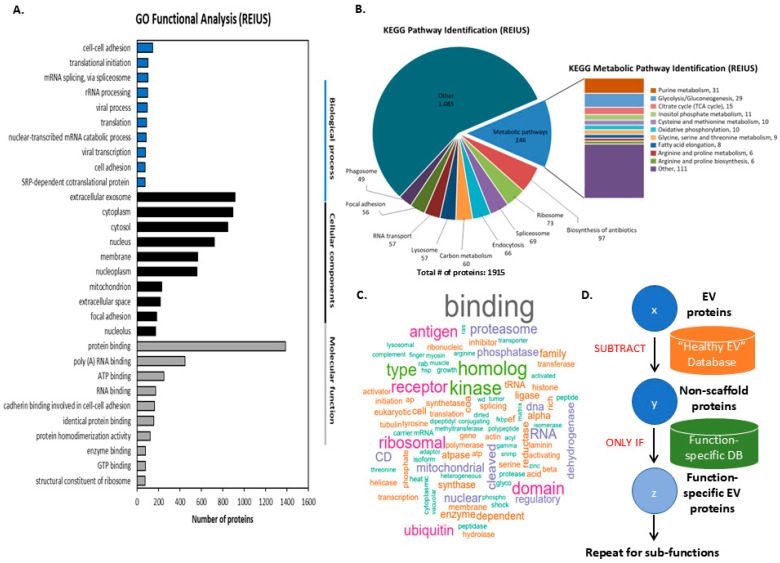
Functional annotation of proteins isolated from REIUS-derived extracellular vesicles. (**A**) Gene Ontology (GO) annotation of identified proteins categorized into biological process, cellular component, and molecular function. Enriched categories include protein binding, exosomal localization, and nucleic acid-associated functions. Statistical significance was determined using DAVID’s modified Fisher’s exact test with Benjamini–Hochberg correction for multiple testing (FDR < 0.05). (**B**) KEGG pathway analysis of identified proteins, highlighting predominant involvement in metabolic pathways, ribosome function, and biosynthesis of antibiotics, with further subcategorization of metabolic processes. Pathways with a false discovery rate (FDR) < 0.05, adjusted by the Benjamini–Hochberg method, were considered statistically significant. (**C**) Word cloud representation highlights the most frequent molecular functions and protein classes among the dataset, emphasizing binding, receptor, kinase, and ribosomal proteins. Word colors are for visual clarity only and do not represent any specific meaning. (**D**) The DFS workflow for identifying function-specific extracellular vesicle (EV) proteins. EV protein candidates (x) are filtered by subtracting proteins found in a “Healthy EV” reference database to yield non-scaffold proteins (y). These are then cross-referenced with a function-specific database to identify function-specific EV proteins (z). The process can be iteratively repeated for sub-functions.

Next, we analyzed the proteomic cargo of melanoma exosomes isolated from Mel888, chosen for its aggressive metastatic profile and extensive data on immune response characterization [30]. Gene Ontology (GO) functional analysis revealed a diverse array of biological processes, cellular components, and molecular functions in the associated proteins identified from these exosomes. (Figure 2A). The most prominent biological processes identified included cell–cell adhesion, translational initiation, mRNA splicing, rRNA processing, and viral processes. GO analysis highlights the roles of melanoma exosome proteins in intercellular communication and the regulation of gene expression. Cellular component analysis revealed strong enrichment for extracellular exosome-associated proteins, alongside proteins localized to the cytoplasm, cytosol, nucleus, membrane, and nucleoplasm, reflecting the diverse subcellular origins of exosomal cargo. The presence of mitochondrial and nucleolar proteins further highlights the broad intracellular representation within exosomes. In terms of molecular function, protein binding was the most prevalent category, followed by poly(A) RNA binding, ATP binding, and RNA binding, suggesting a predominance of proteins involved in molecular interactions and nucleic acid regulation. Additional functions such as enzyme binding, GTP binding, and structural roles within ribosomes were also observed, emphasizing the multifaceted contributions of melanoma exosomal proteins to cellular processes and tumor progression. Potentially, subclassifying “oncogenic driver proteins” may allow capturing of proteins that are more directly implicated in driving tumor progression, distinguishing them from proteins with broader roles in metastasis or cellular function to provide functional clarity of proteins that are subsequently focused upon.

KEGG pathway analysis of the 1915 proteins identified from Mel888-derived exosomes revealed their involvement in diverse cellular pathways and metabolic processes (Figure 2B). The majority of proteins (1085) were classified as “Other,” representing pathways not specifically categorized within the major KEGG classifications. Among the defined pathways, metabolic pathways represented the largest category with 246 proteins, followed by biosynthesis of antibiotics (97 proteins), ribosome (73 proteins), spliceosome (69 proteins), endocytosis (66 proteins), and carbon metabolism (60 proteins). Additional pathways included lysosome (57 proteins), RNA transport (57 proteins), focal adhesion (56 proteins), and phagosome (49 proteins). Detailed analysis of the metabolic pathway subset revealed enrichment in fundamental cellular processes, with purine metabolism being the most represented (31 proteins), followed by glycolysis/gluconeogenesis (29 proteins), citrate cycle/TCA cycle (15 proteins), and Inositol phosphate metabolism (11 proteins). Other metabolic processes included cysteine and methionine metabolism, oxidative phosphorylation, glycine/serine/threonine metabolism, fatty acid elongation, arginine/proline metabolism and biosynthesis, collectively highlighting the metabolic complexity of melanoma exosome cargo and its potential role in reprogramming recipient cell metabolism.

The prominent representation of glycolytic and metabolic pathways in melanoma exosome proteomics data provides compelling justification for examining acidification-related processes mediated by these vesicles. Of particular significance, the identification of 29 proteins involved in glycolysis/gluconeogenesis, along with proteins from the tricarboxylic acid (TCA) cycle (15 proteins). Melanoma exosomes may deliver complex enzymatic payloads capable of disrupting the tightly regulated expression or function of key tricarboxylic acid (TCA) cycle enzymes. This disruption can result in the accumulation of unused, acidic TCA intermediates, leading to functional impairment of the TCA cycle, and a metabolic shift toward glycolysis, which is a less intermediate-dependent yet more robust pathway for ATP generation commonly observed to be activated in diseased states [31]. This finding aligns with emerging evidence that tumor acidity and exosome release are strictly interconnected, with acidified tumor environments producing exosomes with enhanced pro-oncogenic properties [13]. Furthermore, melanoma-derived exosomes have been shown to induce metabolic reprogramming in recipient cells, leading to increased aerobic glycolysis and extracellular acidification through the transfer of specific miRNAs and metabolic enzymes [20]. The lactate dehydrogenase-mediated conversion of pyruvate to lactate, coupled with proton secretion via sodium–proton exchangers, represents a key mechanism by which melanoma cells acidify their microenvironment and enhance exosome-mediated tumor progression. Given the central role of metabolic acidification in melanoma progression and the substantial representation of glycolytic and metabolic proteins within our exosomal proteomics dataset, a subset of identified proteins has been classified according to their direct involvement in acidification and metabolic reprogramming pathways. Simultaneously, analysis of the melanoma exosome proteome revealed a strong enrichment of immune-associated terms, as identified by keywords in UniProt’s functional descriptions (Figure 2C), indicating a distinct immune-related payload. The frequent appearance of the term “antigen” highlights proteins involved in antigen processing and presentation—processes critical for immune recognition and evasion. Additionally, “proteasome” emerged as a major category, reflecting the presence of proteins important for antigen processing and MHC class I presentation, both of which are central to immune surveillance. Other co-functional terms related to immune regulation, such as “binding,” “ribosomal,” “kinase,” “homolog,” “domain,” and “ubiquitin,” also appeared frequently. Given that melanoma exosomes are known to carry immunosuppressive molecules (e.g., PD-L1) that facilitate immune evasion [22], the prevalence of immune-related functional terms further supports the classification of these exosomes as a distinct immune-modulating subset. Taken together, melanoma exosomal proteins can be delineated into three functional subsets according to their involvement in distinct biological pathways: acidification/metabolic, immune modulation, and oncogenic driver proteins.

To systematically identify functionally relevant exosomal proteins apart from highly abundant, constitutively expressed exosomal protein scaffold carried by the vesicular payload, we propose a proteomic workflow that can be referred to as “Scaffold-free Functional Deconvolution” (SFD), as illustrated in Figure 2D. Briefly, the initial set of exosomal protein candidates (denoted as x) is filtered by subtracting proteins present in a comprehensive “healthy exosome” reference database, resulting in a subset of non-scaffold proteins (y) that are more likely to be associated with disease or altered states. This refined protein set is then cross-referenced with a curated function-specific database to further isolate proteins (z) with established or predicted roles in the function of interest. This stepwise approach enables the identification of function-specific exosomal proteins, and the workflow can be iteratively applied to delineate sub-functions or additional biological processes as needed. This SFD-based strategy enhances the specificity and relevance of candidate protein selection for downstream validation and mechanistic studies.

### 3.3. Finding Clinically Relevant Biomarkers on Melanoma Exosomes Using SFD

**Figure 3:**
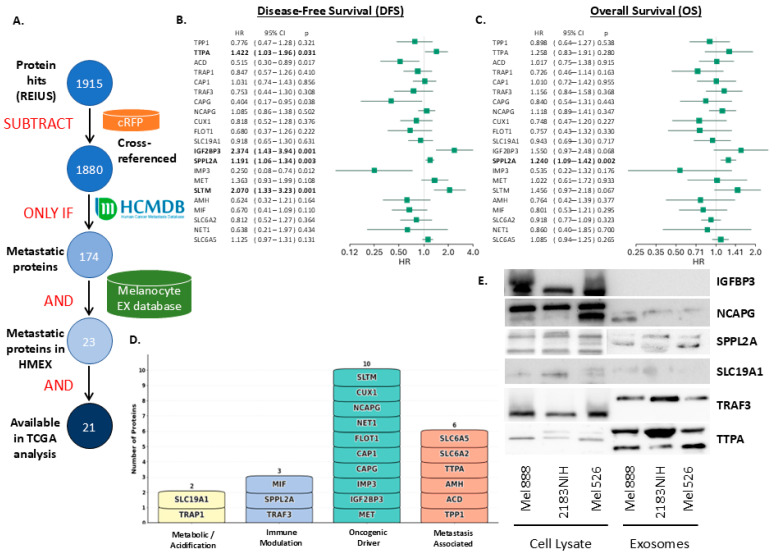
Systematic identification and survival analysis of metastatic proteins from REIUS-derived extracellular vesicles. (**A**) Filtering workflow for protein identification, beginning with 1915 REIUS protein hits and culminating in 21 metastatic proteins available for TCGA analysis through sequential database filtering. (**B**,**C**) Kaplan–Meier survival analysis showing hazard ratios and 95% confidence intervals for disease-free survival (DFS) and overall survival (OS), respectively. (**D**) Functional classification of the 21 proteins into metabolic/acidification, immune modulation, oncogenic driver, and unknown metastatic categories. (**E**) Western blot of proteins with commercially available antibodies confirms biochemically the presence of some of the 21 proteins identified through SFD.

To identify clinically relevant metastatic proteins within the melanoma exosomal cargo, we implemented SFD that refined our initial 1915 protein hits from Mel888 melanoma exosomes through multiple validation steps (Figure 3A). After removing cross-referenced serum proteins and applying the Human Cancer Metastasis Database (HCMDB) filter, 174 metastatic proteins were retained. By further cross-referencing with the melanocyte expression database created using melanocyte exosomes, the number of proteins identified was reduced to 23 proteins, of which 21 are available for survival analysis using TCGA data. In the TCGA melanoma dataset, there are 103 patients who underwent resection for the primary tumor. The patient demographics were shown in Table S1. Median disease-free survival (DFS) was not reached, and median overall survival (OS) was 28.8 months (Figure S1). Univariate analysis revealed that high expression of TTPA, IGF2BP3, SPPL2A, and SLTM estimated poorer DFS (Figure 3B) and that high expression of SPPL2A also estimated poorer OS (Figure 3C). Proteins such as TPP1, TTPA, and ACD demonstrated hazard ratios exceeding 1.0 for both survival metrics, indicating poor prognostic associations, while others, like TRAP1 and GAP1, showed protective effects with hazard ratios below 1.0. Functional classification of these 21 proteins was classified into four distinct categories based on GO terms, KEGG pathway, and Uniprot functional descriptions: metabolic/acidification regulators (*n* = 2), immune modulators (*n* = 3), oncogenic drivers (*n* = 10), and metastasis-associated factors (*n* = 6) (Figure 3D). This systematic approach identified a refined set of EV proteins with both metastatic relevance and clinical prognostic value, providing potential targets for immunotherapeutic intervention. Next, we attempted to determine if the proteomic analysis results in actual proteins present in exosomes by Western blot (Figure 3E). As expected, most proteins identified through SFD were present in both the melanoma parental cell lines and also in exosomes, indicating that the proteomic data connect directly to biochemical results and that the proteins identified here are valid targets for metastatic biomarkers of exosomes that are not otherwise found in normal melanocyte exosomes.

### 3.4. Using SFD to Identify Immunosuppressive Surface Biomarkers

**Figure 4:**
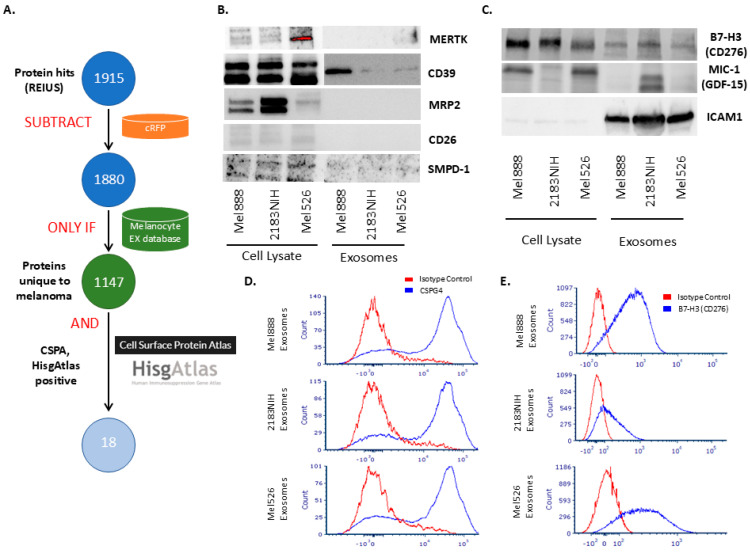
Boolean pipeline-based identification and validation of immunosuppressive biomarkers on melanoma-derived extracellular vesicles. (**A**) Schematic of the stepwise filtering process integrating REIUS proteomics, cRFP subtraction, melanoma specificity, cell surface localization (CSPA), and immunosuppressive function (HisgAtlas), resulting in 18 candidate proteins. (**B**,**C**) Immunoblot analysis of metastasis-associated and predicted EV surface proteins in cell lysates and exosomes from three melanoma cell lines. (**D**) Flow cytometry analysis of CSPG4 surface expression on exosomes, demonstrating robust detection across all lines. (**E**) Flow cytometry analysis of CD276 expression on exosomes, confirming the presence of this immunosuppressive marker on the surface of exosomes.

To identify immunosuppressive proteins that are expressed on the surface of melanoma exosomes, we applied an SFD-based pipeline that systematically refined our initial dataset of 1915 protein hits through sequential database interrogations (Figure 4A). After subtracting serum proteins using the cRFP database, 1880 proteins remained for further analysis. Application of the melanocyte exosome database filter identified 1147 proteins unique to melanoma exosomes, effectively eliminating constitutive vesicle components shared with healthy melanocytes. Cross-referencing with the Cell Surface Protein Atlas (CSPA) reduced this to 120 surface-accessible proteins, which were subsequently filtered through the HisgAtlas immunosuppression database to yield 18 candidate biomarkers with documented immunoregulatory functions: APOH, B7-H3 (CD276), CALR, CD26, CD39, CD44, CSF1, FSTL1, GDF15, GPNMB, GRN, ICAM1, L1CAM, MERTK, MICB, MRP2, SMPD-1, and TIMP1 that were detected on melanoma exosomes but absent from melanocyte-derived exosomes in our dataset.

We selected a subset of proteins in which antibodies were commercially available (Figure 4B). Western blot analysis revealed that all examined proteins unique to melanoma based on mass spectroscopy were detectable in the parental melanoma cell lines; however, most of these membrane proteins were not observed in the corresponding melanoma-derived exosomes. This discrepancy is likely attributable to the limited sensitivity of Western blotting in detecting partially hydrophobic, poorly soluble, and low-abundance membrane proteins, which is several orders of magnitude less sensitive than mass spectrometry-based detection methods. B7-H3 (CD276), MIC-1, and ICAM1, however, were detectable and present in all three melanoma exosomes (Figure 4C).

To assess the presence of surface proteins on melanoma-derived exosomes, we first employed CSPG4, a well-established surface marker for melanoma exosomes, using a bead-based flow cytometry assay. CSPG4 was eliminated from SFD as it was present in melanocyte exosomes, possibly in very minute quantities. Briefly, exosomes isolated from melanoma cell lines were captured on flow cytometry beads coated with an anti-CSPG4 antibody, then surface expression of CSPG4 on these beads was bound with melanoma exosomes overnight and detected using a secondary, non-competing anti-CSPG4 antibody recognizing a distinct epitope, as per the reported technique [32]. Flow cytometric analysis demonstrated robust CSPG4 signal on exosomes from all three melanoma cell lines tested (Mel888, 2183NIH, and Mel526), as indicated by a clear shift relative to isotype control (Figure 4D). These results confirmed the presence of CSPG4 on the surface of melanoma exosomes and validated the specificity of our detection approach. We attempted the same approach with B7-H3 (CD276) and obtained less distinct but similar positive results (Figure 4E). However, several attempts to demonstrate the presence of MIC-1 (GDF-15) and ICAM-1 using flow cytometry did not yield sufficiently consistent results to report.

Our findings, based on direct mass spectrometric comparison, provide further evidence that the exosomal surface proteome is shaped by malignant transformation and may serve as a source of novel biomarkers or therapeutic targets in melanoma.

### 3.5. Meta-Analysis of Vesiclepedia Using SFD Approach

To test the meta-analytic capability of SFD, we explored the use of SFD to interrogate a large EV database available, such as Vesiclepedia. Vesiclepedia’s unparalleled repository of 566,911 protein entries across 3533 extracellular vesicle (EV) studies makes it a formidable testbed for SFD in digesting and eliminating systematically the ubiquitous scaffold EV proteins while focusing on precise oncogenic (e.g., melanoma EV) cargo protein payload identity. By leveraging Vesiclepedia’s healthy EV proteome datasets combined into an “healthy EV” database, SFD can be tested to examine this massive compendium into functionally stratified outputs for examining clinically actionable targets while mitigating immunogenicity risks from non-pathological proteins circulating extracellularly in healthy individuals.

**Figure 5:**
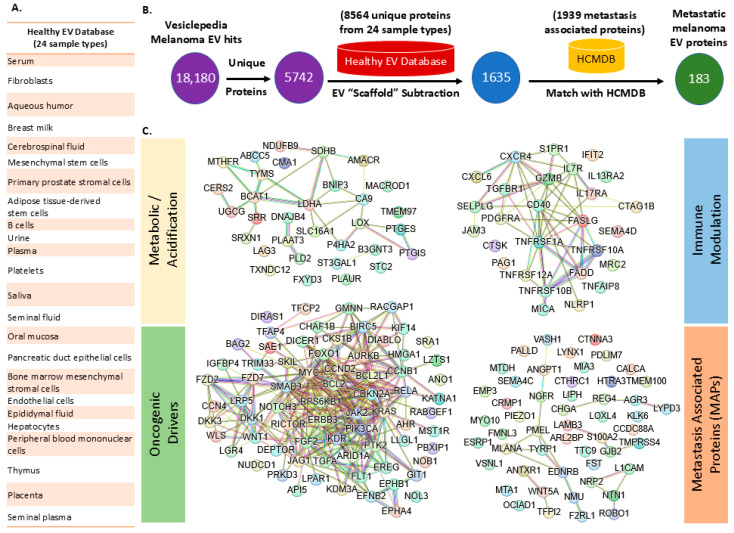
Systematic identification and network categorization of metastatic melanoma extracellular vesicle (EV) proteins. (**A**) Compilation of a comprehensive Healthy EV Database from 24 normal human sample types to define baseline EV protein content. (**B**) Stepwise filtering pipeline: melanoma EV proteomics data are filtered to remove healthy “scaffold” proteins and cross-referenced with the Human Cancer Metastasis Database (HCMDB), resulting in 183 metastatic melanoma EV proteins. (**C**) STRING network analysis of these 183 proteins, functionally grouped into metabolic/acidification, oncogenic drivers, immune modulation, and metastasis-associated protein categories, reveals distinct interaction clusters underlying metastatic processes in melanoma. Edges in the STRING network are color-coded to indicate the type of supporting evidence: green lines represent gene neighborhood, red lines indicate gene fusions, blue lines show gene co-occurrence, purple lines denote experimental evidence, yellow lines indicate text mining, light blue (sky blue) lines represent database annotation, and black lines indicate co-expression.

Beginning with 18,180 proteins identified in Vesiclepedia melanoma EV datasets, we subtracted 8564 proteins present across 24 healthy EV sample types, including serum, fibroblasts, cerebrospinal fluid, and tissue-specific sources such as breast milk and aqueous humor (Figure 5A,B). This “EV scaffold subtraction” reduced the dataset to 1635 melanoma-enriched proteins, which were subsequently cross-referenced with the Human Cancer Metastasis Database (HCMDB) containing 1939 metastasis-associated proteins. The final SFD pipeline yielded 183 metastatic melanoma EV proteins that are both functionally relevant to cancer progression and absent from healthy EV cargo. Network analysis of these 183 proteins revealed distinct functional clusters, including metabolic acidification regulators, oncogenic signaling drivers, and immune modulators (Figure 5C). This approach addresses a critical challenge in EV-based therapeutic development by systematically removing proteins that are present only in metastasis-specific targets. The SFD methodology represents a paradigm shift from traditional discovery workflows, enabling identification of EV cargo that drives pathological processes without the confounding influence of constitutive vesicular components that may compromise both therapeutic specificity and immunological safety profiles.

## 4. Discussion

There have been several attempts made to determine the exact payload of these exosomes that originate from a dedicated biogenesis pathway through the proteomic (or equivalent) analyses of TEX derived from melanoma [33], bladder cancer [34], epithelial ovarian cancer [35], prostate cancer [36], pancreatic cancer [37] and breast cancer [38]. Recent advances in exosome isolation techniques, such as REIUS (a novel method for isolating high-purity exosomes), or similar techniques, have enabled deeper insights into their functional roles by providing exosomes of superior purity, reduced contamination, and preserved structural integrity, while remaining compatible with downstream analysis for direct proteomic analysis. This study explores how melanoma-derived exosomes, enriched with specific protein cargo, can reprogram neighboring cells to fulfill the metabolic and survival needs of metastatic tumors. In particular, human melanoma-derived exosomes have been found to increase metastasis and promote immunosuppression in the TME and to contain PD-L1 as a relevant biomarker of clinical response [39]. Our previous report determined that less than 1% of melanoma exosomes are positive for surface PD-L1, indicating that the payload of these melanoma exosomes is remarkably heterogeneous and complex [20]. Despite their nanosized scale, the potential for exosomes to carry a diverse array of biomolecules beyond immune checkpoints is more apparent than before.

However, generating consistent evidence across analytical platforms with vastly different sensitivities presents a significant challenge. First, the high-resolution sensitivity of MS/MS Orbitrap proteomics allows for the detection of low-abundance EV proteins that may be undetectable by conventional Western blotting, especially when antibody affinity or epitope accessibility is limited. As a result, proteins identified by mass spectrometry may not always be possible to validate by immunodetection methods, emphasizing the importance of considering methodological limitations when interpreting proteomic data. Second, the inability to detect these proteins by Western blot may reflect technical limitations, such as insufficient antibody sensitivity to post-translational modification or epitope masking due to EV-specific post-translational modifications or conformational changes. Notably, this phenomenon has been observed with canonical EV markers, where mass spectrometry confirms their presence, but antibody-based detection remains inconsistent.

Additionally, our Western blot data on novel proteins identified through the SFD pipeline relied on antibodies targeting rare proteins that have only recently become commercially available and for which extensive validation and optimization are still ongoing. As a result, some variability in signal response may occur, potentially impacting the robustness and reproducibility of protein detection and quantification. Ongoing improvements in antibody development and validation are expected to strengthen future studies involving these uncommon protein targets. Overall, our data suggest that protein expression in parental cells does not always predict EV cargo abundance, reinforcing the need for direct and sensitive EV profiling to accurately capture their molecular landscape.

SFD operationalizes an evolutionary principle intrinsic to biological systems: deconvolution of functional activities through elimination. This mirrors how pattern recognition receptors (PRRs) detect pathogen-associated molecular patterns (PAMPs), except that here, the “pathogen” is a released vesicle. This approach has not been systematically applied to cancer EVs, a gap originating from the historical view of cancer as a dysregulated “self” rather than a foreign entity. However, vesicles shed by cancer cells act as functionally independent vectors of pathology. Although pathogens display conserved signatures, cancer EV encode a dynamic repertoire of tumor-specific signals that evade traditional classification frameworks and still hold potential as unique identifiers of malignancy. By adopting this pathology-discriminatory logic, SFD transforms bulk cancer EV data into clinically actionable targets, revealing how melanoma co-opts intercellular communication pathways to drive metastasis. Proteins detected using this method are typically found in exosomes derived from melanocytes under standard conditions, i.e., meaning they are naturally produced by human cells during normal physiological function. However, finding these same proteins inside circulating EVs from healthy individuals is unusual and could signal the presence of a potentially disease-causing cargo. The detection of these proteins in EVs, rather than in their conventional cellular compartments, reflects an aberrant localization that signals a disruption of homeostasis. This ectopic EV association thus represents a disease-specific signature, distinguishing pathological states from normal cellular physiology and supporting the use of EV proteomics for biomarker discovery in cancer. By focusing on EV-associated proteins that meet these functional criteria, this pipeline captures key mediators of intercellular communication within the tumor ecosystem, providing a foundation for understanding resistance mechanisms and immunogenic potential. This approach ultimately enables the identification of biomarkers that can withstand the rigors of clinical validation while maintaining biological significance in melanoma progression and therapeutic response.

As a methodology, SFD is able to reveal well-known proteins in melanoma exosomes that are immune-related and are also surface-expressed proteins, such as CD26 (DPP4), CD44, CALR, B7-H3 (CD276), CSF1, ICAM1, L1CAM, MICB, APOH, TIMP1, and CD39. These molecules are widely recognized for their roles in cancer biology and immune regulation. For instance, CD44 and ICAM1 are key adhesion molecules implicated in tumor cell migration, metastasis, and modulation of the immune response, with ICAM1 upregulation on melanoma cells and exosomes linked to enhanced metastatic potential and immune escape [40,41]. Of note is a high saturation of ICAM1 in melanoma exosomes relative to the cell lysate. This phenomenon is also observed in other studies [41]. CD26 (DPP4) and CALR are involved in immune modulation and cell signaling, while B7-H3 (CD276) is a prominent immune checkpoint molecule highly expressed in melanoma and other tumors, contributing to immunosuppression and tumor progression [42,43]. CSF1, L1CAM, and MICB are also associated with immune evasion, cell adhesion, and metastatic dissemination. CSF1 is a key regulator of the tumor microenvironment, promoting the differentiation and accumulation of immunosuppressive tumor-associated macrophages (TAMs) and correlating with disease progression and resistance to immune checkpoint blockade in melanoma. Ref. [44] L1CAM and MICB are similarly implicated in processes that facilitate tumor cell adhesion, immune escape, and metastatic spread, underscoring their roles as drivers of melanoma progression and contributors to both immune evasion and metastatic potential [45]. The presence of these proteins in melanoma exosomes highlights their potential role in shaping the tumor microenvironment and facilitating immune escape, consistent with previous reports that melanoma-derived exosomes carry immunosuppressive and pro-metastatic factors.

Proteins such as GDF15, MERTK, MRP2, SMPD-1, GPNMB, GRN, and FSTL1, while less widely recognized, have emerging roles in cancer progression, immune modulation, and metabolic regulation. MIC-1 (GDF15) and GRN are associated with tumor-induced immune tolerance and inflammation [46,47], while MERTK is a receptor tyrosine kinase involved in the clearance of apoptotic cells and immune suppression [48]. GPNMB and FSTL1 have been linked to tumor invasion, metastasis, and modulation of the extracellular matrix [49,50]. The identification of these moderately and less well-known proteins exclusively in melanoma exosomes suggests that they may contribute to the distinct immunosuppressive and pro-metastatic phenotype of melanoma, warranting further investigation into their specific functions and potential as therapeutic targets.

These proteins may shed light on how melanoma may drive metastasis through its EV payload. Analogous to the observation that colorectal cancer (CRC) cell-derived exosomal B7-H3 can be internalized by human umbilical vein endothelial cells (HUVECs), leading to activation of the AKT/mTOR/VEGFA signaling axis and enhanced migratory and angiogenic potential, it is plausible that melanoma-derived exosomes may similarly confer new functional properties to recipient cells. This suggests that exosomal transfer of specific proteins or signaling molecules from melanoma cells could induce phenotypic shifts in the tumor microenvironment or in distant tissues exposed to these vesicles [51].

In parallel, a comprehensive meta-analysis of Vesiclepedia melanoma EV data confirms the well-established TME-modulating proteins. Immune-modulating proteins such as CXCR4, CD40, IL7R, TGFBR1, and SEMA4D are frequently targeted for cancer intervention studies. Furthermore, the presence of well-studied oncogenic driver proteins such as ERBB3, PIK3CA, BIRC5, WNT1, and DKK1 reaffirms the need for greater research focus on these molecules. Lastly, the well-established oncogenes KRAS and MYC were also identified within melanoma-derived EV, supporting a potential role for EV-mediated transfer of these tumorigenic factors in promoting malignant transformation and tumor progression.

A key limitation of the SFD approach that it operates on the presence or absence of proteins, rather than their quantitative abundance. This binary framework can lead to the exclusion of biologically relevant proteins that are present at low levels in control samples but are highly enriched in disease states. As an illustration, CSPG4, which is a well-established melanoma exosome surface marker, was excluded from the SFD-derived list because it was detected in melanocyte exosomes as well, and was confirmed using Western blot. According to the Vesiclepedia database, CSPG4 is also found in many healthy cell types and also a constituent in saliva and urine EV. This illustrates a broader challenge with SFD and related feature detection techniques: an underestimation of their biological significance. As a result, SFD is valuable for identifying unique features from a large proteomic database, but it may overlook important quantitative differences that are well established in the literature and relevant to disease biology. This limitation highlights the need to complement SFD with robust quantitative analyses and literature-based curation to fully capture the purpose of comparing exosome proteomes in cancer and control samples. Yet, SFD does serve its purpose in raising thought-provoking questions. For example, while CD44 is an abundant protein in all cells, including melanocytes [52], it was puzzlingly undetectable in normal melanocyte exosomes for all four donors, yet present in malignant melanoma-derived exosomes. This paradoxical pattern also aligns with the absence of CD44 in melanocyte exosomes reported in Vesiclepedia, which includes an in-depth proteomic study of 1843 proteins from uveal melanocyte exosomes [53].

CD44, through alternative splicing and interaction with the tumor microenvironment, is not merely a marker of cancer-initiating cells but also actively contributes to tumor progression by facilitating cell migration, niche preparation, epithelial-mesenchymal transition, and resistance to apoptosis. Particularly, CD44 variant isoforms (CD44v), often upregulated in malignant cells, endow cancer-initiating cells such as self-renewal, niche preparation, epithelial–mesenchymal transition, and resistance to apoptosis. It can thus be hypothesized that melanoma exosomes, by exporting CD44 and its variants, can induce profound changes in recipient cells and the microenvironment, ultimately facilitating metastatic dissemination and immune evasion [40]. In a sense, this possibility expands upon the potential of using proteomics not for peptide matching of physiologically accurate proteins but of variants that can initiate cancer. SFD relied on a peptide database derived from UniProt, which predominantly contains canonical human protein sequences and does not account for disease-specific variants, neoantigens, or fusion proteins. Therefore, the identification of exosomal proteins was restricted to those annotated in standard databases. Fusion proteins and neoantigenic peptides, increasingly recognized as hallmarks of cancer, may be present in melanoma-derived EV and contribute to their pathogenic signaling [54,55]. Such an approach would enhance the resolution of exosomal proteomic profiling, facilitate the discovery of novel biomarkers for interventional use, and yield deeper insights into the molecular mechanisms underpinning melanoma progression and immune evasion [56]. The meta-analysis of the entire Vesiclepedia melanoma EV protein payload, yielding a manageable 183 proteins, provides a valuable starting point to examine for unique peptide sequences to identify EV-based neoantigen markers. Therefore, a future study reanalyzing the existing mass spectrometry data using updated peptide databases that include neoantigen and fusion protein sequences could reveal additional, disease-specific exosomal markers.

## 5. Conclusions

Melanoma-derived EVs represent a critical yet underexplored axis of metastatic disease. Leveraging SFD, our comprehensive analysis delineates a distinct exosomal molecular signature associated with melanoma. ICAM-1 was markedly overexpressed in melanoma-derived exosomes, implicating it in the facilitation of tumor-associated intercellular adhesion and metastatic dissemination. Proteomic profiling further identified the enrichment of SPPL2A in the melanoma exosomal cargo (a molecule previously correlated with diminished disease-free and overall survival) suggesting its potential utility as a prognostic indicator within the extracellular vesicle compartment. Notably, CD276 emerged as a selective surface marker, uniquely characterizing melanoma exosomes and presenting a compelling target for diagnostic stratification and therapeutic intervention. Additionally, CD44 was readily detected in melanoma exosomes but conspicuously absent from those originating from normal melanocytes, reinforcing the tumor-specificity of exosomal biomarker profiles. Collectively, these findings elucidate key components of the melanoma exosomal proteome and highlight the translational promise of extracellular vesicle-associated markers for refined melanoma prognosis and targeted therapy. These results lay the groundwork for future translational studies targeting EV-associated proteins in melanoma, with the eventual aim of advancing therapeutic strategies for metastatic disease.

## Data Availability

The data supporting the findings of this study are available from the corresponding author upon reasonable request. Publicly archived datasets (Vesiclepedia, HisgAtlas, HCMDB, cRFP Repository, CBioportal) were analyzed during this study. Where applicable, data sharing is subject to privacy or ethical restrictions.

## Supplementary Materials

The following supporting information can be downloaded at: https://www.mdpi.com/article/10.3390/cancers17152509/s1, Figure S1: Median OS and OFS; Figure S2: Uncropped images; Table S1: Patient demographics of TCGA melanoma cohort.
