# Supplementary material for: Scaffold-Free Functional Deconvolution Identifies Clinically Relevant Metastatic Melanoma EV Biomarkers
Source: Cancers (Basel). 2025 Jul 30;17(15):2509. doi: 10.3390/cancers17152509 (PMC12345765; doi:10.3390/cancers17152509)

Scaffold-free Functional Deconvolution Identifies Clinically Relevant Metastatic Melanoma EV Biomarkers

Full, uncropped Western blots corresponding to

[Figure 1C] CD63 and ß-actin
and
[Figure 4C] MIC-1 (GDF15) and B7-H3(CD276)

in the main manuscript, provided as per editorial request

*Corresponding authors for manuscript:

Shin La Shu and Marc Ernstoff

Email: shushinla@gmail.com

[Figure 1C] CD63

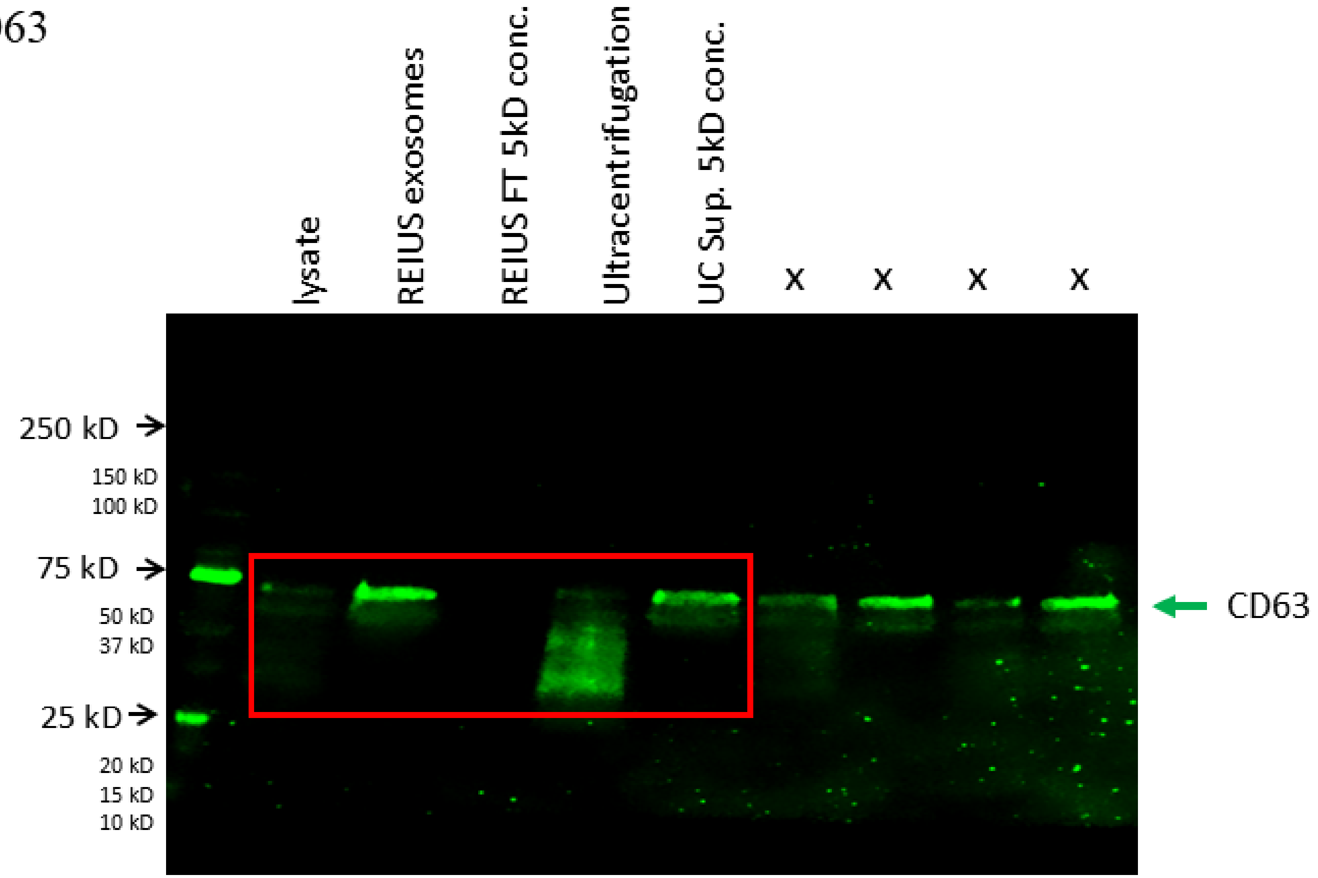

Densitometry Analysis and Intensity Ratio of CD63 Western Blot Bands

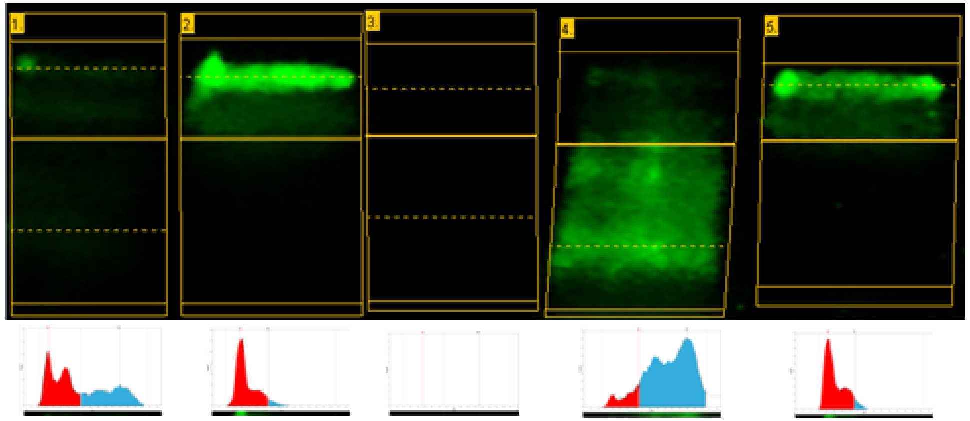

Lane No.	Raw Volume	**CD63 Intensity Ratio**
1	594	1.0
2	2558	4.3
3	0	0.0
4	624	1.1
5	1890	3.2

[Figure 1C] ß-actin

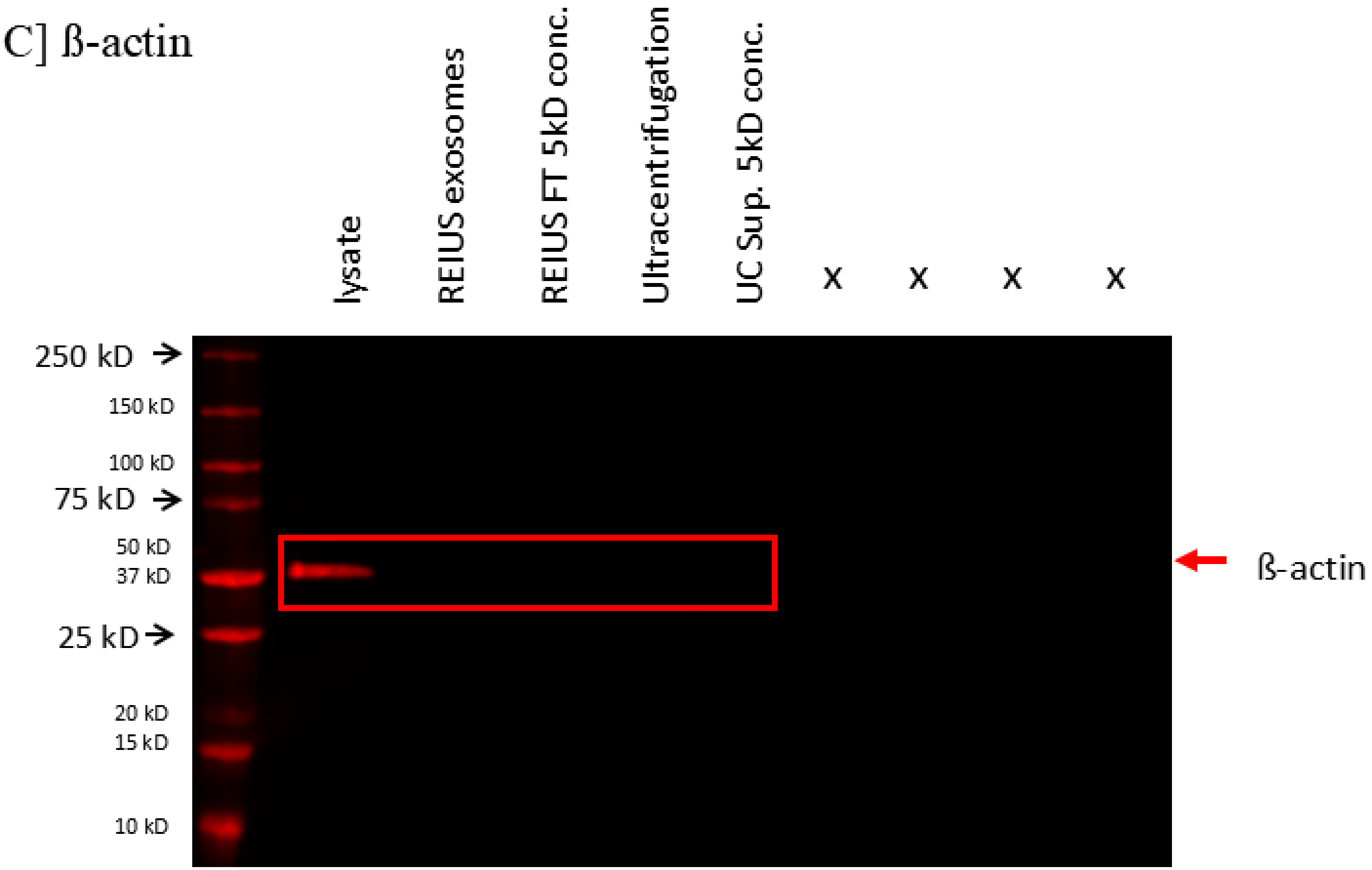

Figure 3E

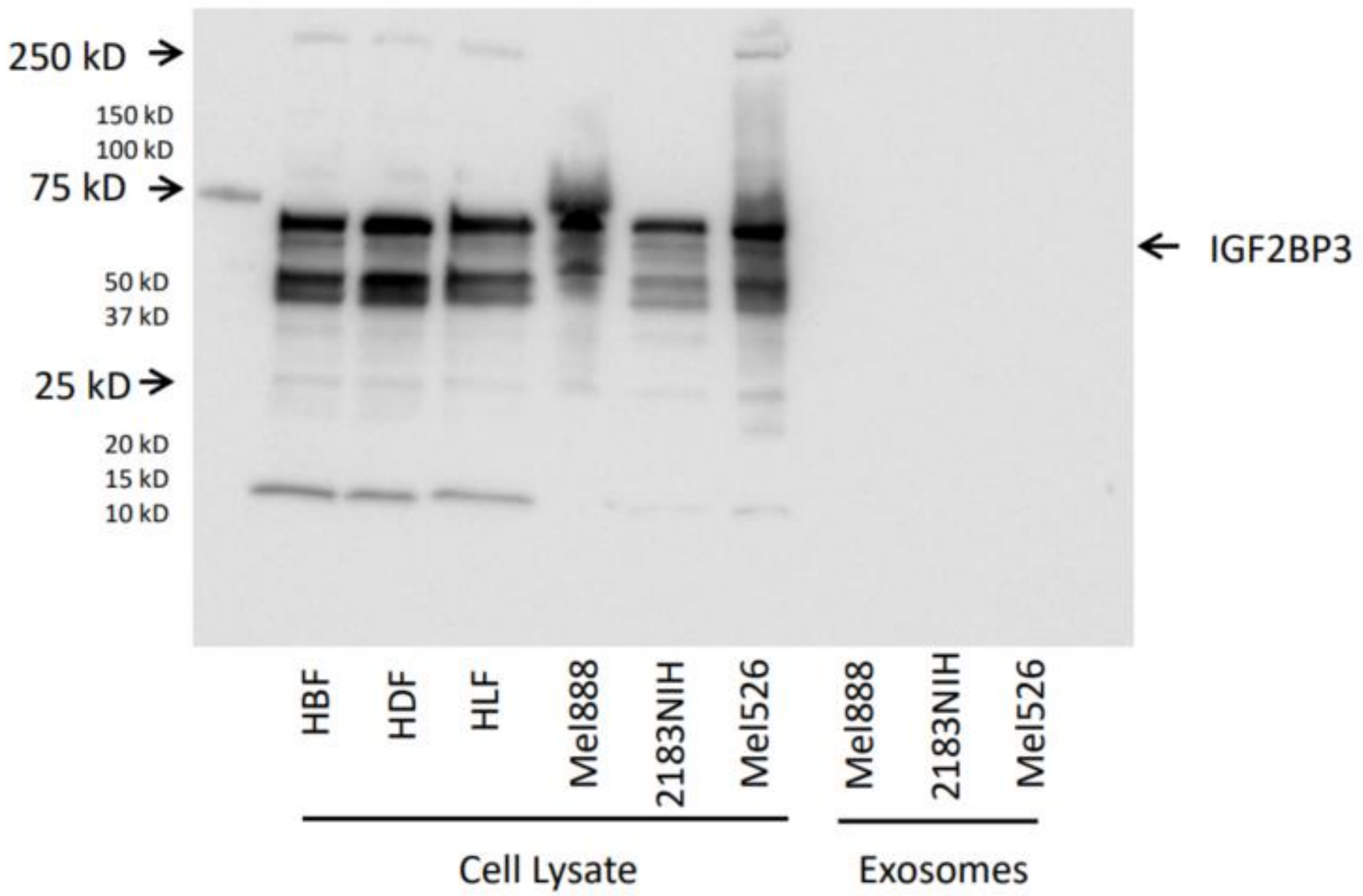
250 kD
150 kD
100 kD
75 kD
50 kD
37 kD
25 kD
20 kD
15 kD
10 kD
IGF2BP3
HBF
HDF
HLF
Mel888
2183NIH
Mel526
Mel888
2183NIH
Mel526
Cell Lysate
Exosomes

Figure 3E

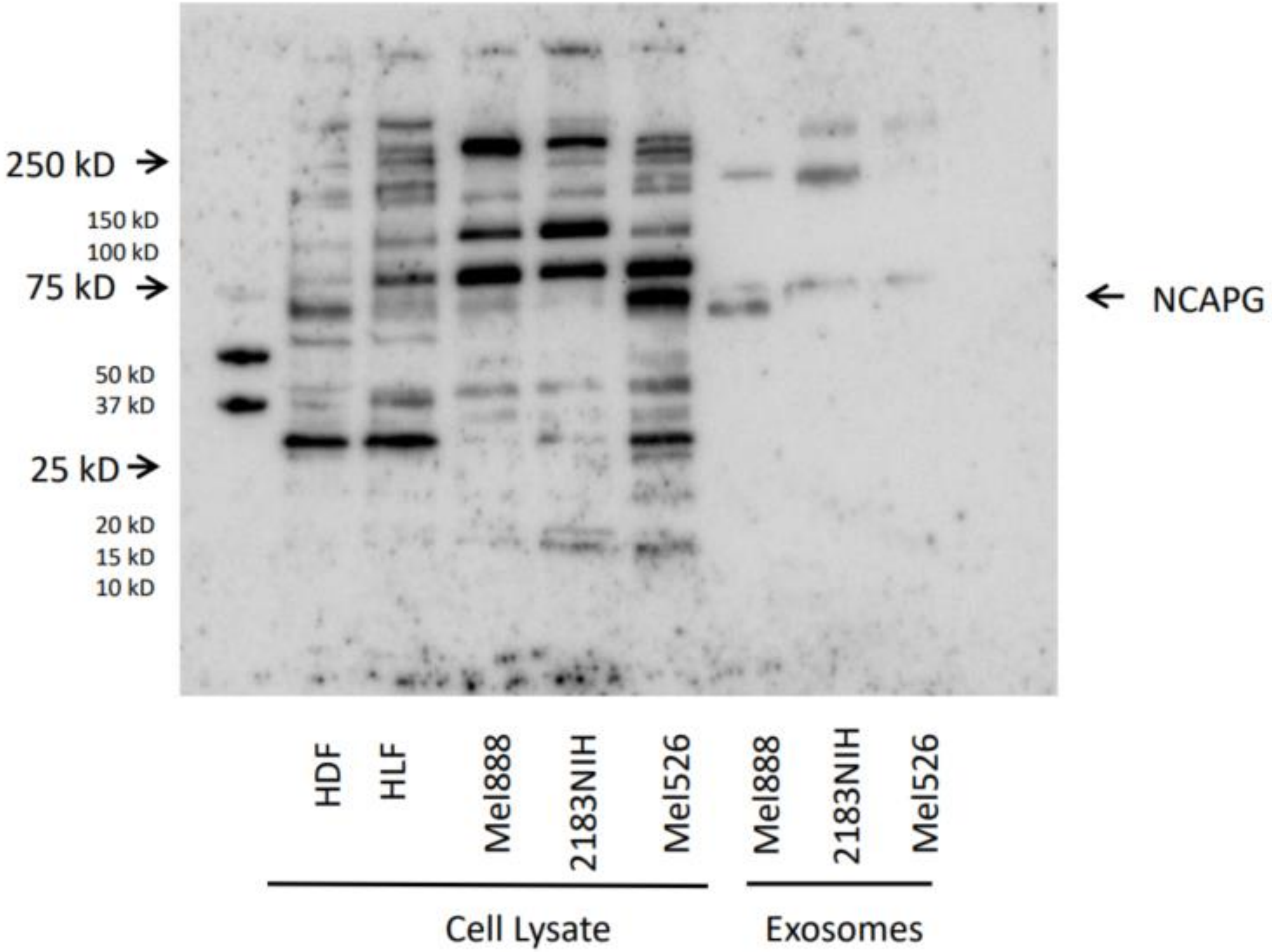
250 kD
150 kD
100 kD
75 kD
50 kD
37 kD
25 kD
20 kD
15 kD
10 kD
NCAPG
HDF
HLF
Mel888
2183NIH
Mel526
Mel888
2183NIH
Mel526
Cell Lysate
Exosomes

Figure 3E

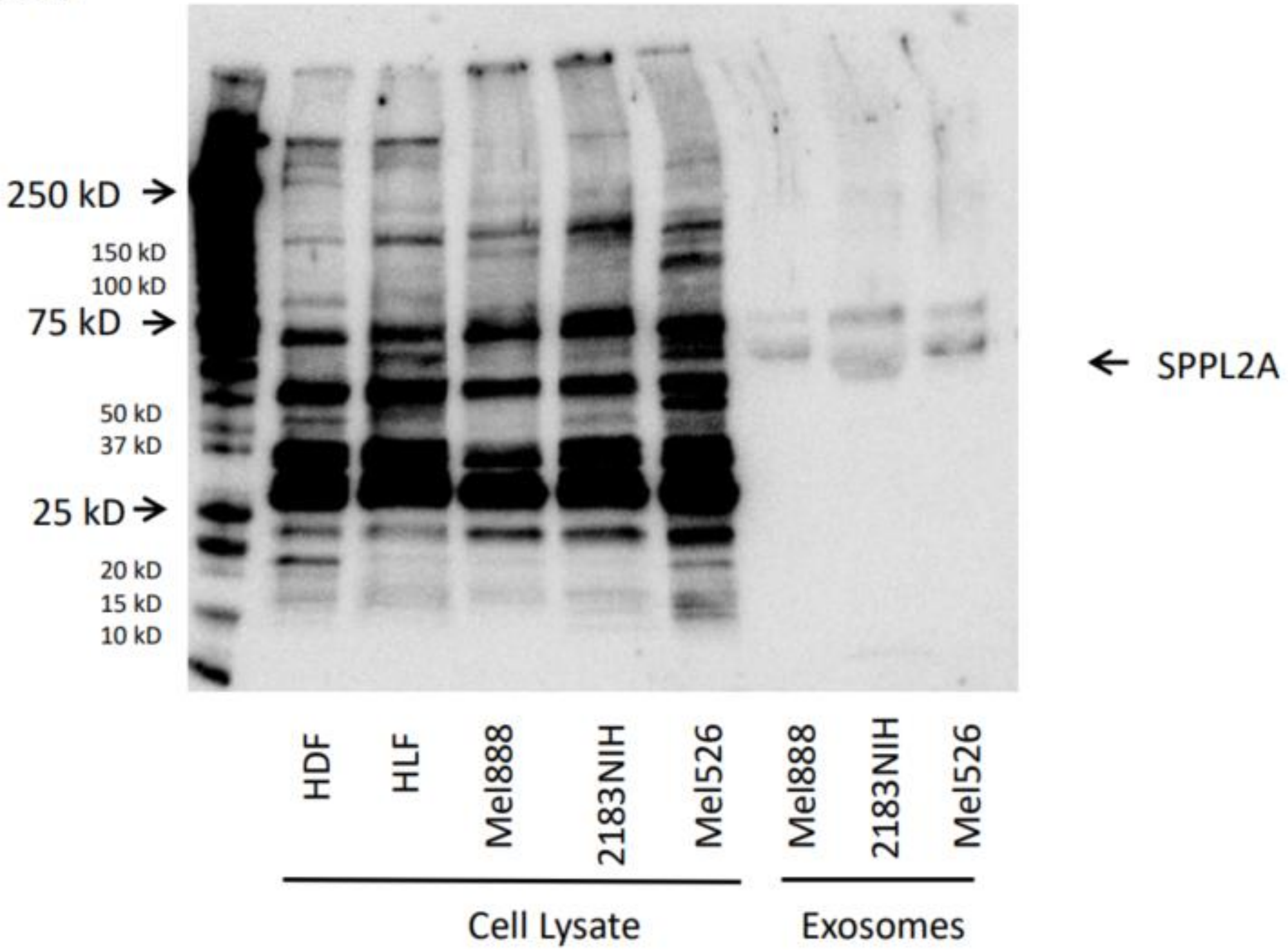
250 kD
150 kD
100 kD
75 kD
50 kD
37 kD
25 kD
20 kD
15 kD
10 kD
SPPL2A
HDF
HLF
Mel888
2183NIH
Mel526
Mel888
2183NIH
Mel526
Cell Lysate
Exosomes

Figure 3E

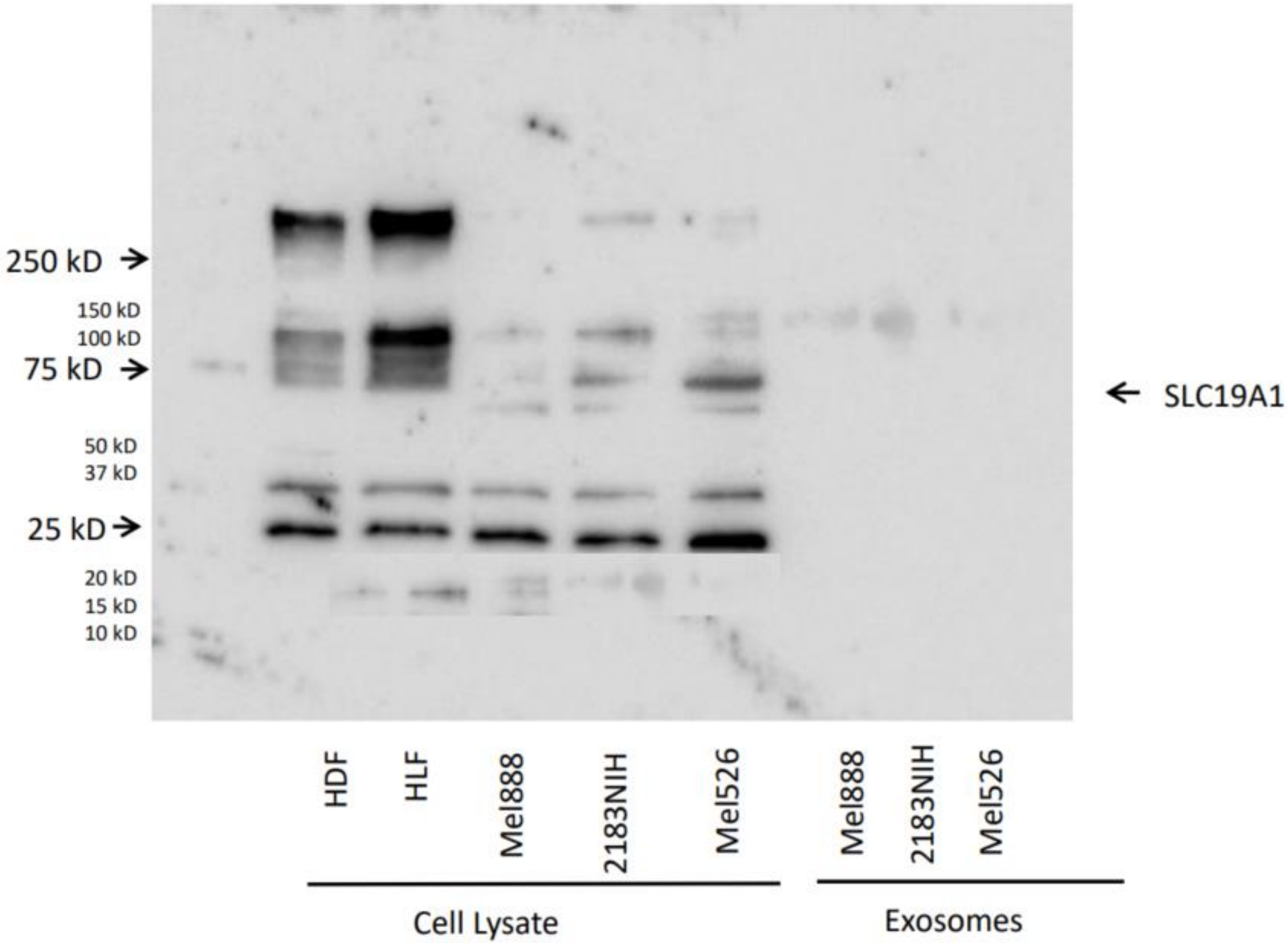
250 kD
150 kD
100 kD
75 kD
50 kD
37 kD
25 kD
20 kD
15 kD
10 kD
SLC19A1
HDF
HLF
Mel888
2183NIH
Mel526
Mel888
2183NIH
Mel526
Cell Lysate
Exosomes

Figure 3E

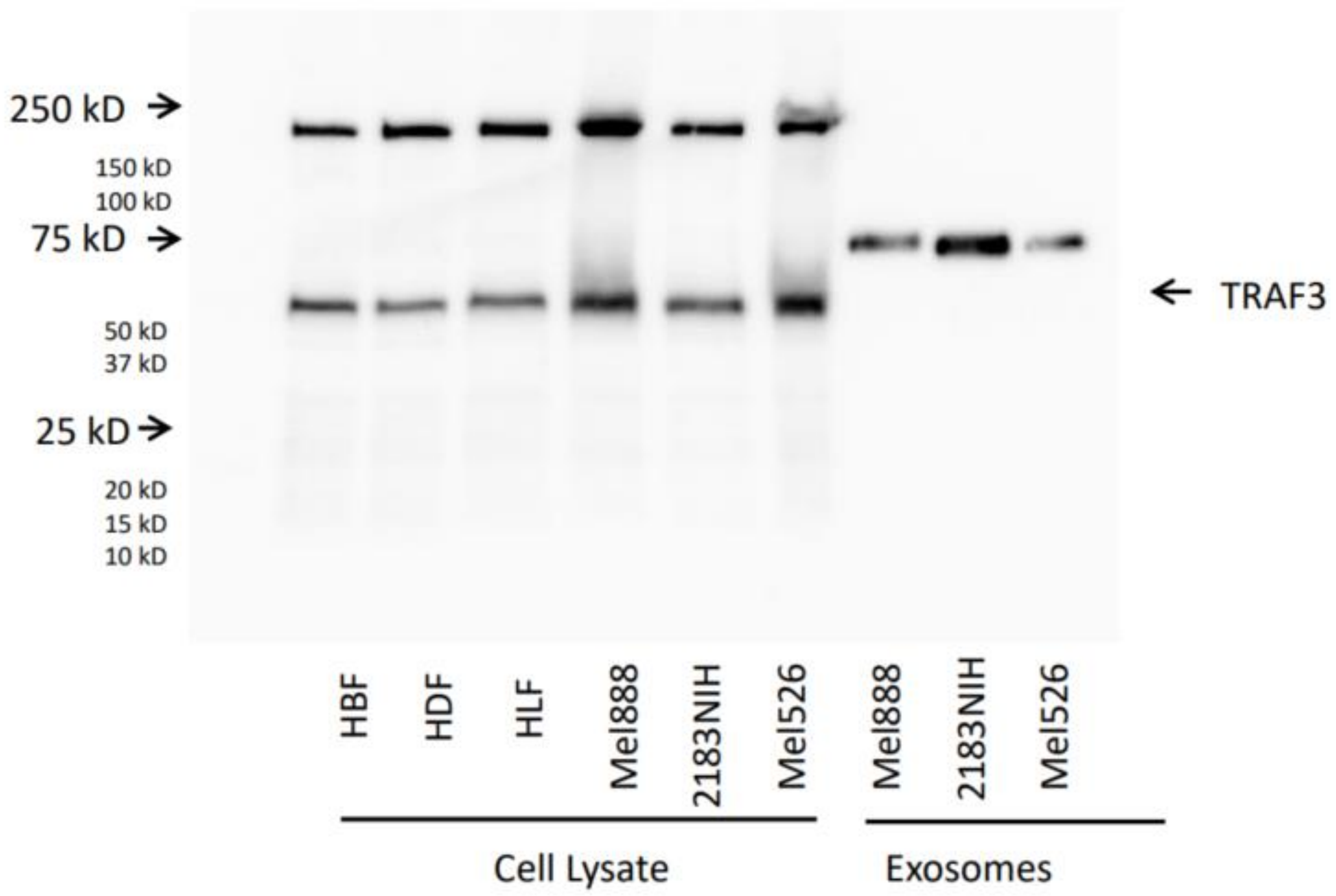
250 kD
150 kD
100 kD
75 kD
50 kD
37 kD
25 kD
20 kD
15 kD
10 kD
TRAF3
HBF
HDF
HLF
Mel888
2183NIH
Mel526
Mel888
2183NIH
Mel526
Cell Lysate
Exosomes

Figure 3E

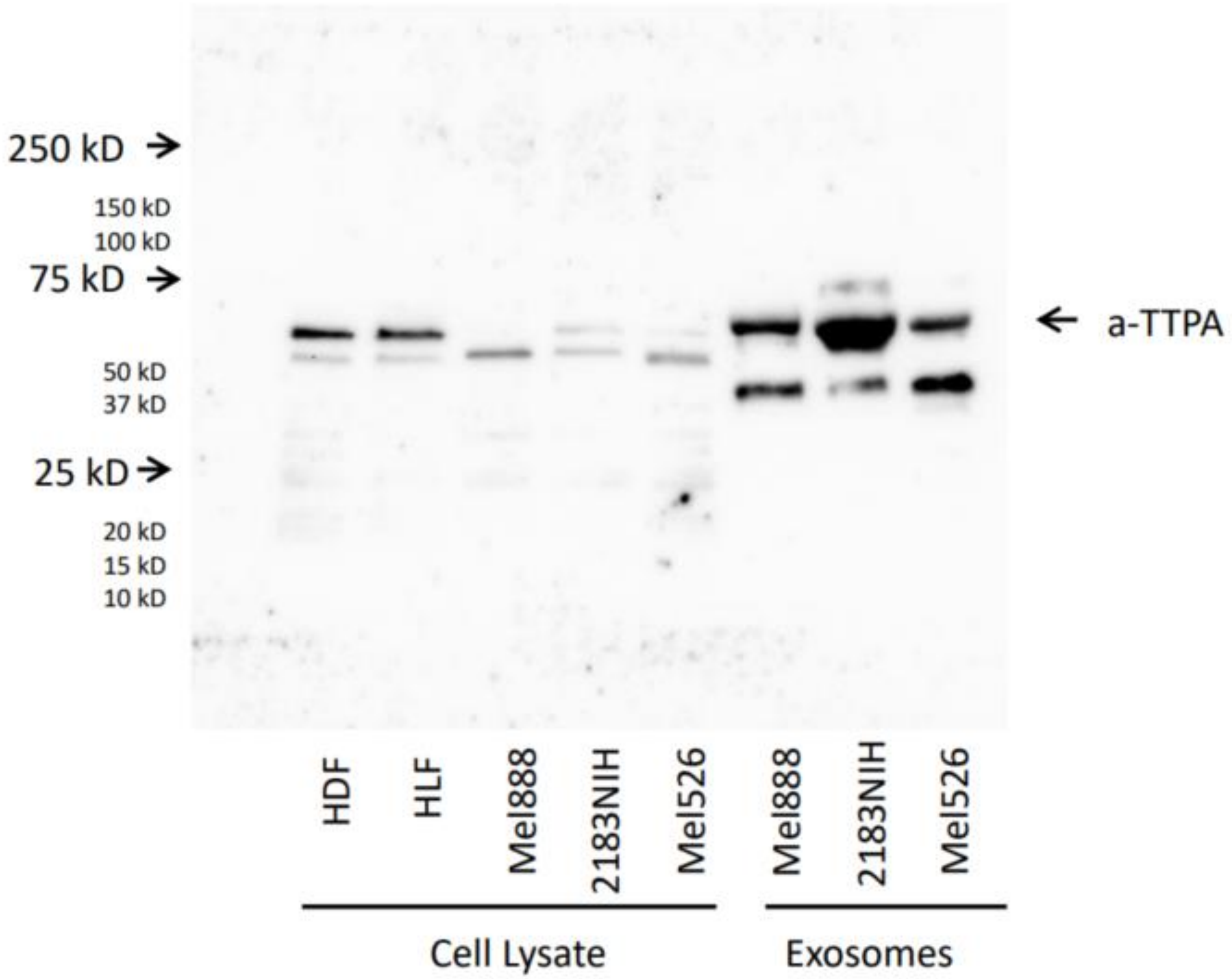
250 kD
150 kD
100 kD
75 kD
50 kD
37 kD
25 kD
20 kD
15 kD
10 kD
a-TTPA
HDF
HLF
Mel888
2183NIH
Mel526
Mel888
2183NIH
Mel526
Cell Lysate
Exosomes

Figure 4B

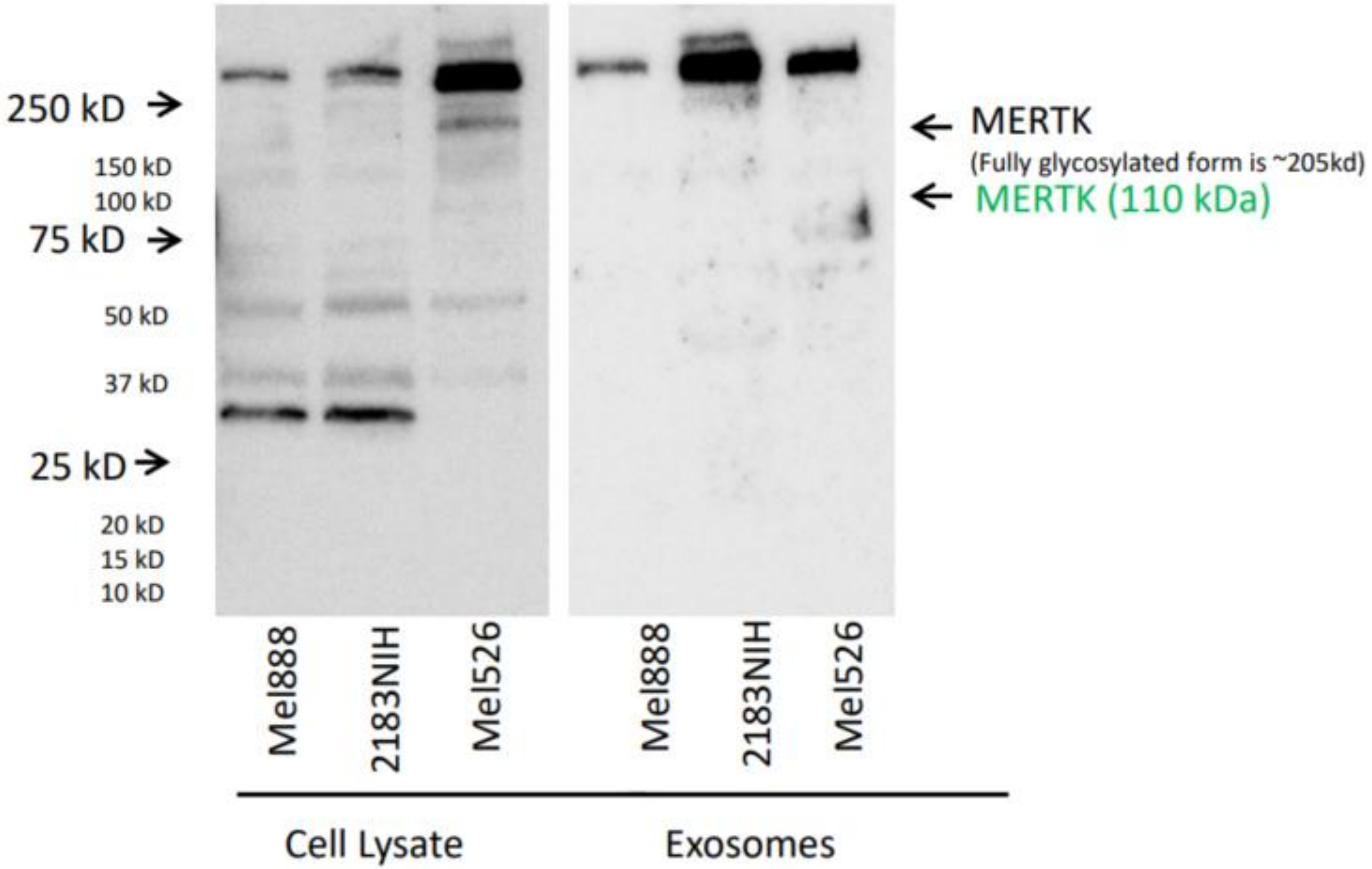
250 kD
150 kD
100 kD
75 kD
50 kD
37 kD
25 kD
20 kD
15 kD
10 kD
MERTK
(Fully glycosylated form is ~205kd)
MERTK (110 kDa)
Mel888
2183NIH
Mel526
Mel888
2183NIH
Mel526
Cell Lysate
Exosomes

Figure 4B

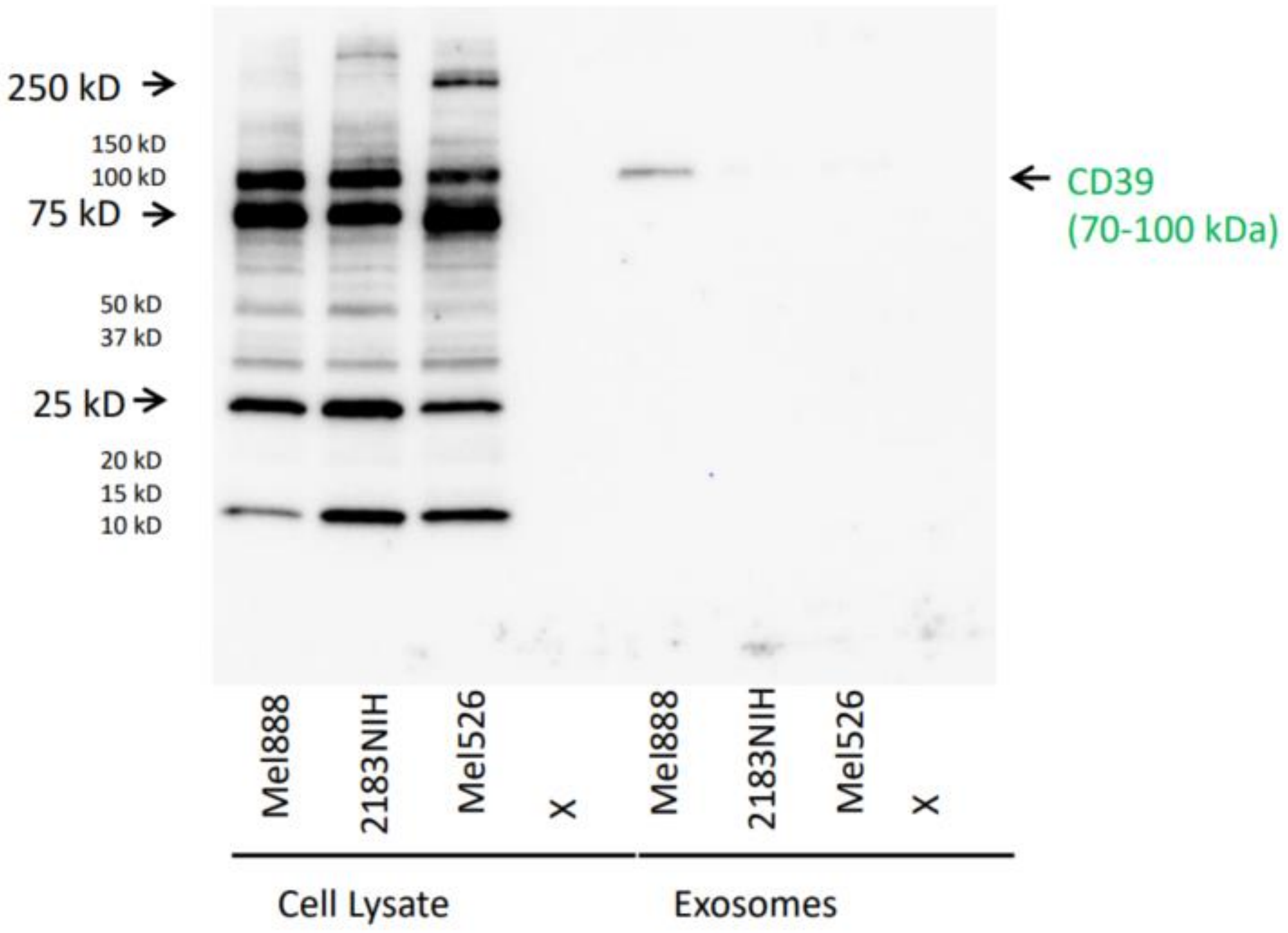

250 kD
150 kD
100 kD
75 kD
50 kD
37 kD
25 kD
20 kD
15 kD
10 kD
CD39
(70-100 kDa)
Mel888
2183NIH
Mel526
X
Mel888
2183NIH
Mel526
X
Cell Lysate
Exosomes

Figure 4B

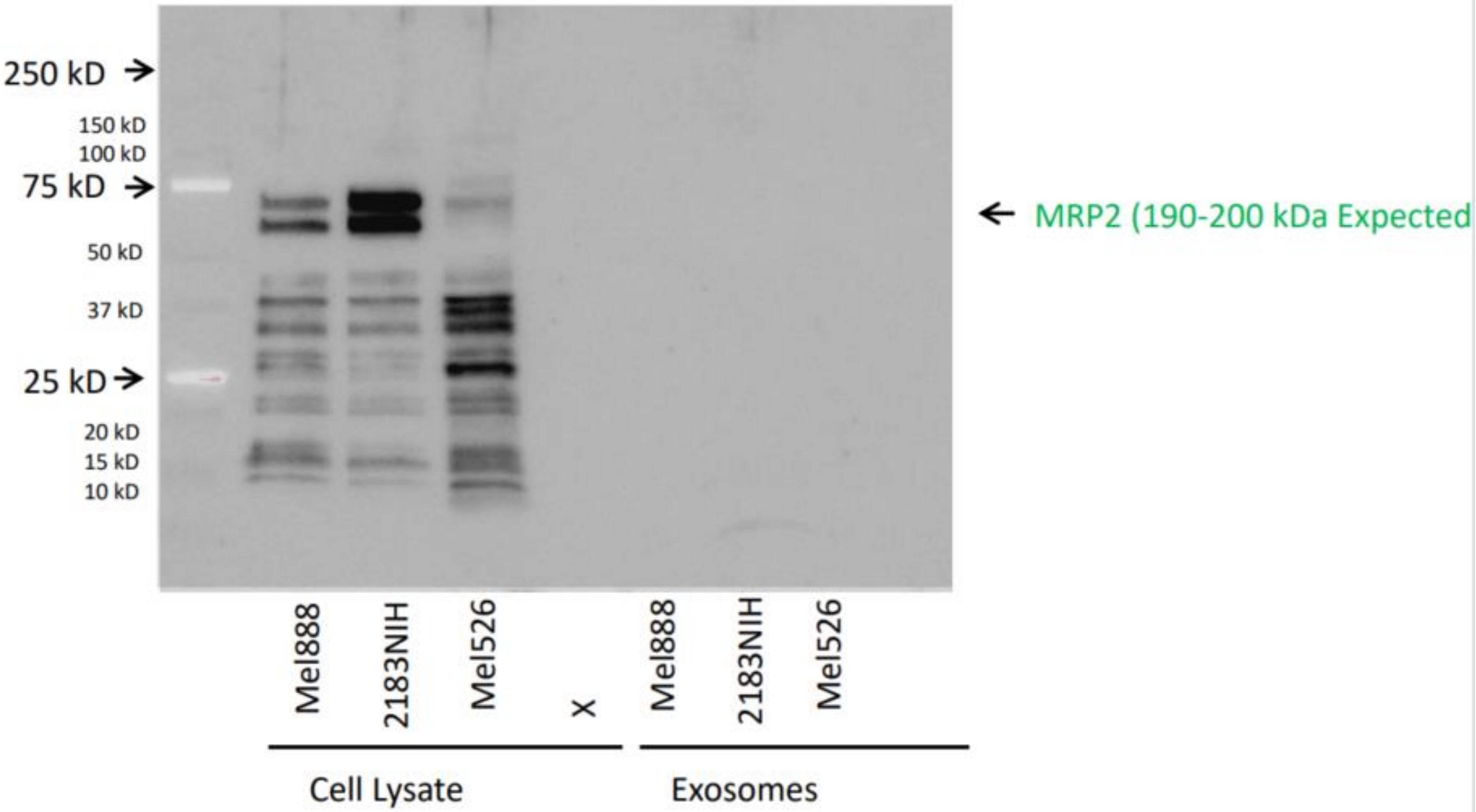

Multidrug resistance-associated protein 2 (**MRP2**) also called canalicular multispecific organic anion transporter 1 (cMOA) or ATP-binding cassette sub-family. ABC proteins transport various molecules across extra- and intra-cellular membranes.

Figure 4B

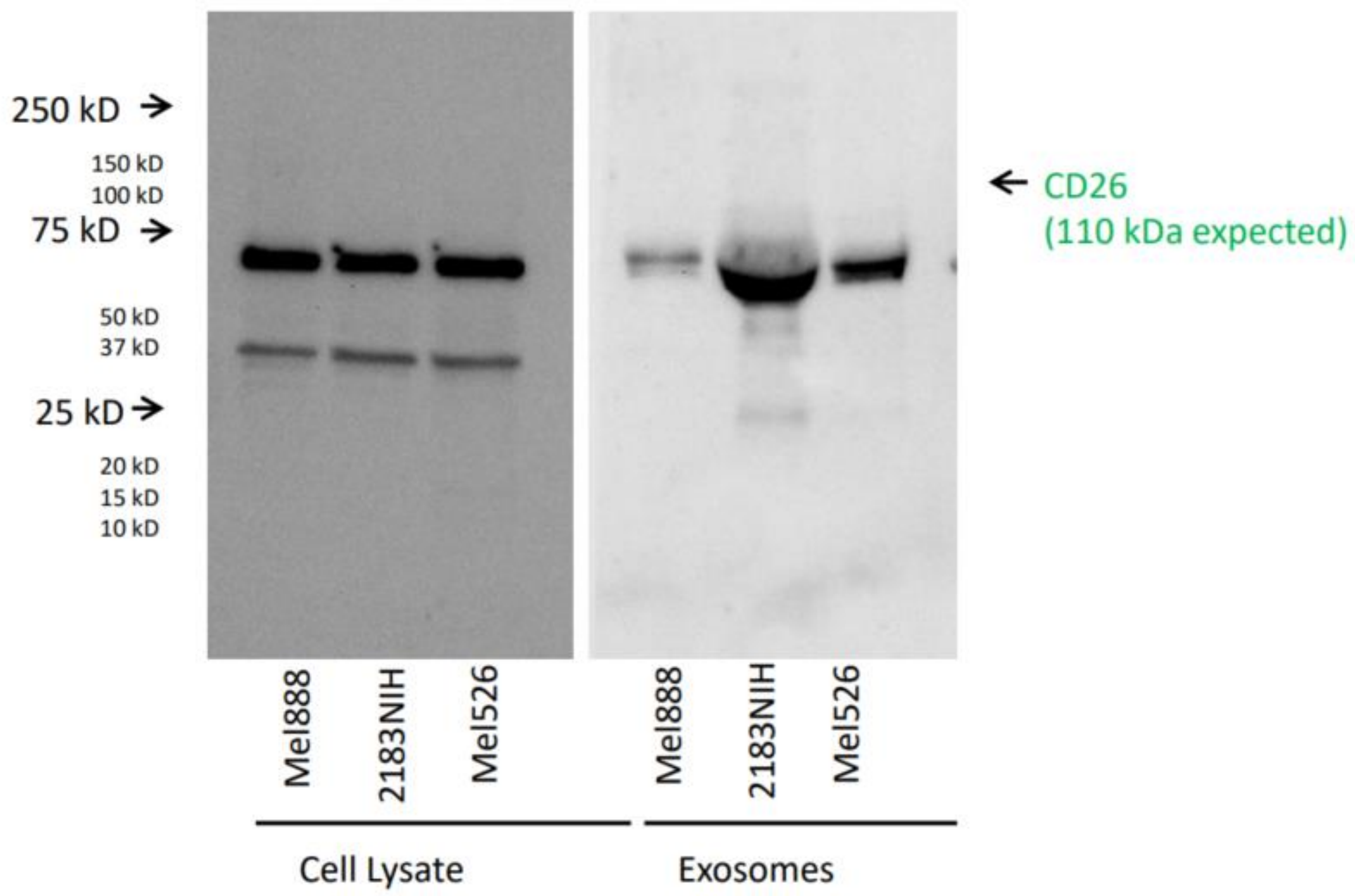
250 kD
150 kD
100 kD
75 kD
50 kD
37 kD
25 kD
20 kD
15 kD
10 kD
CD26
(110 kDa expected)
Mel888
2183NIH
Mel526
Mel888
2183NIH
Mel526
Cell Lysate
Exosomes

Figure 4B

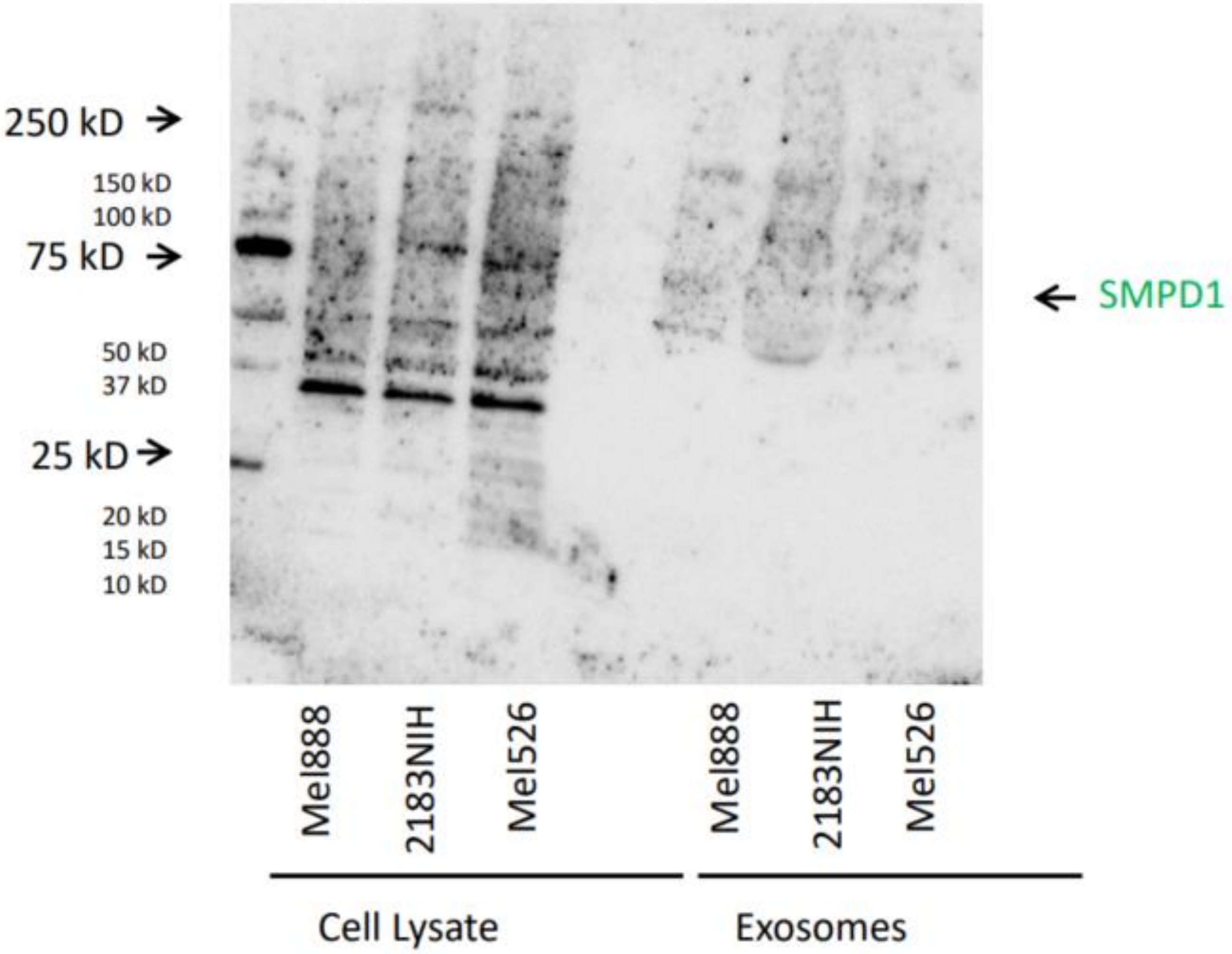
250 kD
150 kD
100 kD
75 kD
50 kD
37 kD
25 kD
20 kD
15 kD
10 kD
SMPD1
Mel888
2183NIH
Mel526
Mel888
2183NIH
Mel526
Cell Lysate
Exosomes

[Figure 4C] B7-H3(CD276)

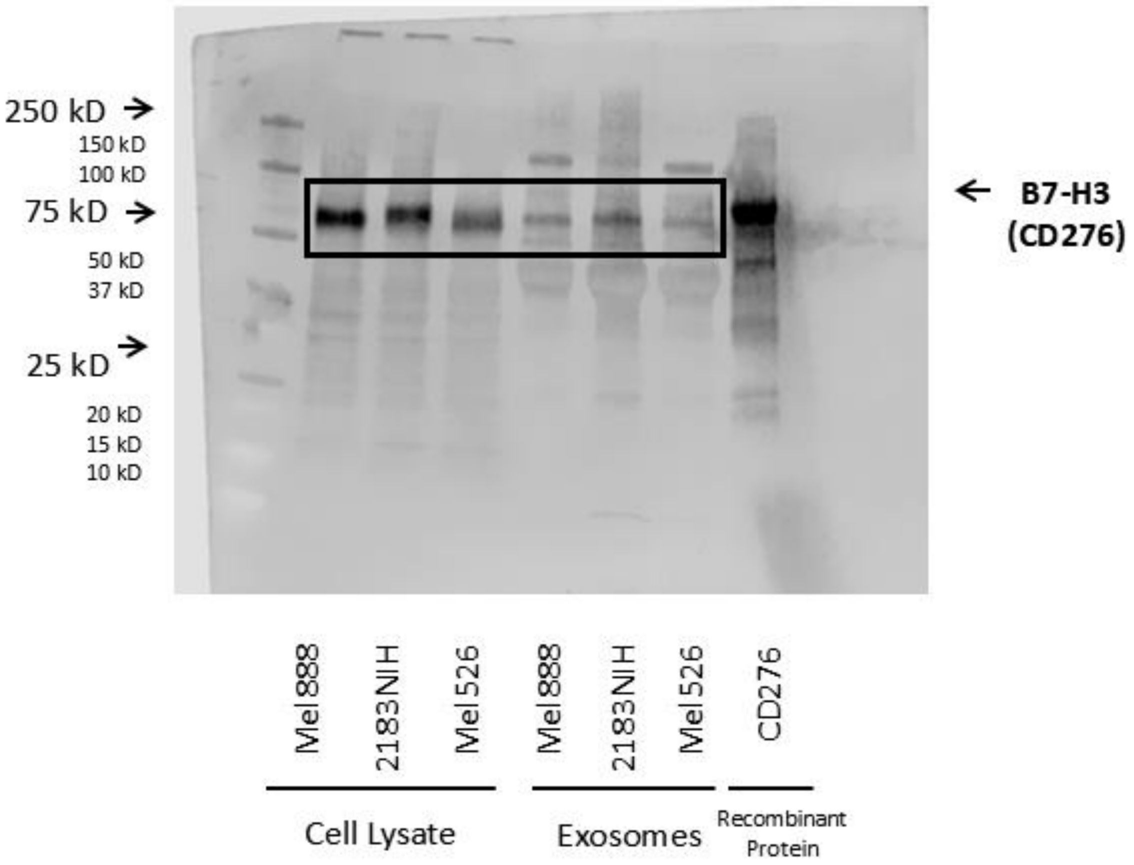

[Figure 4C] MIC-1 (GDF15)

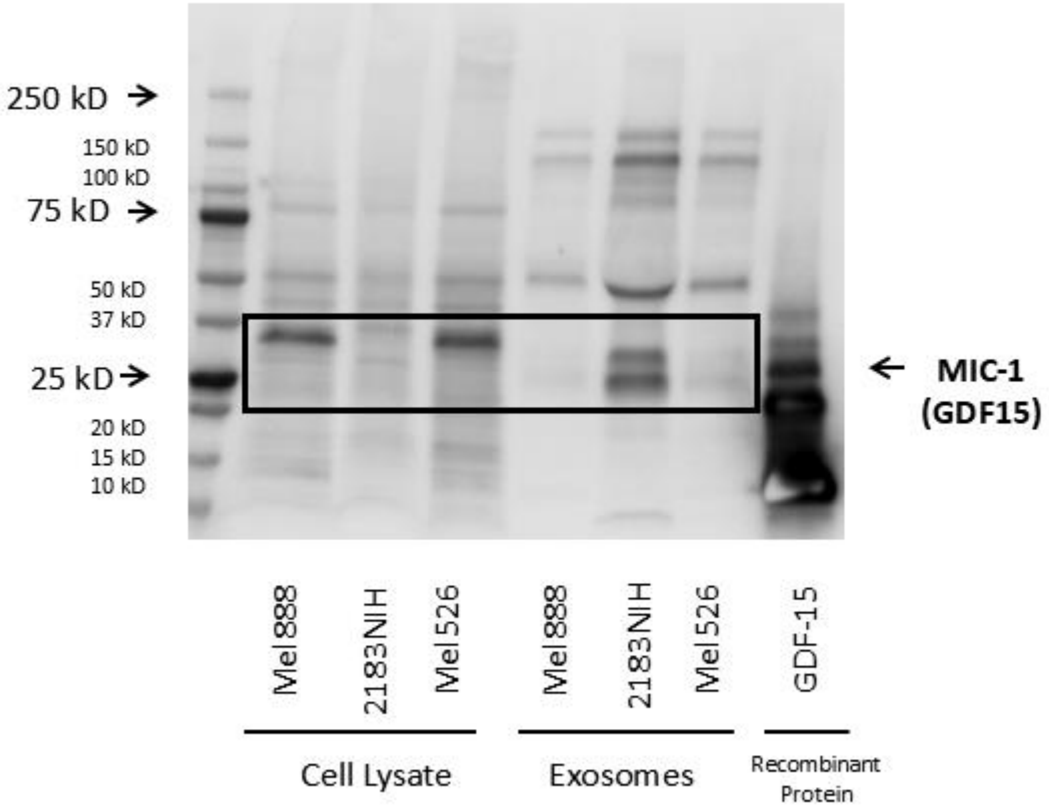

Figure 4C

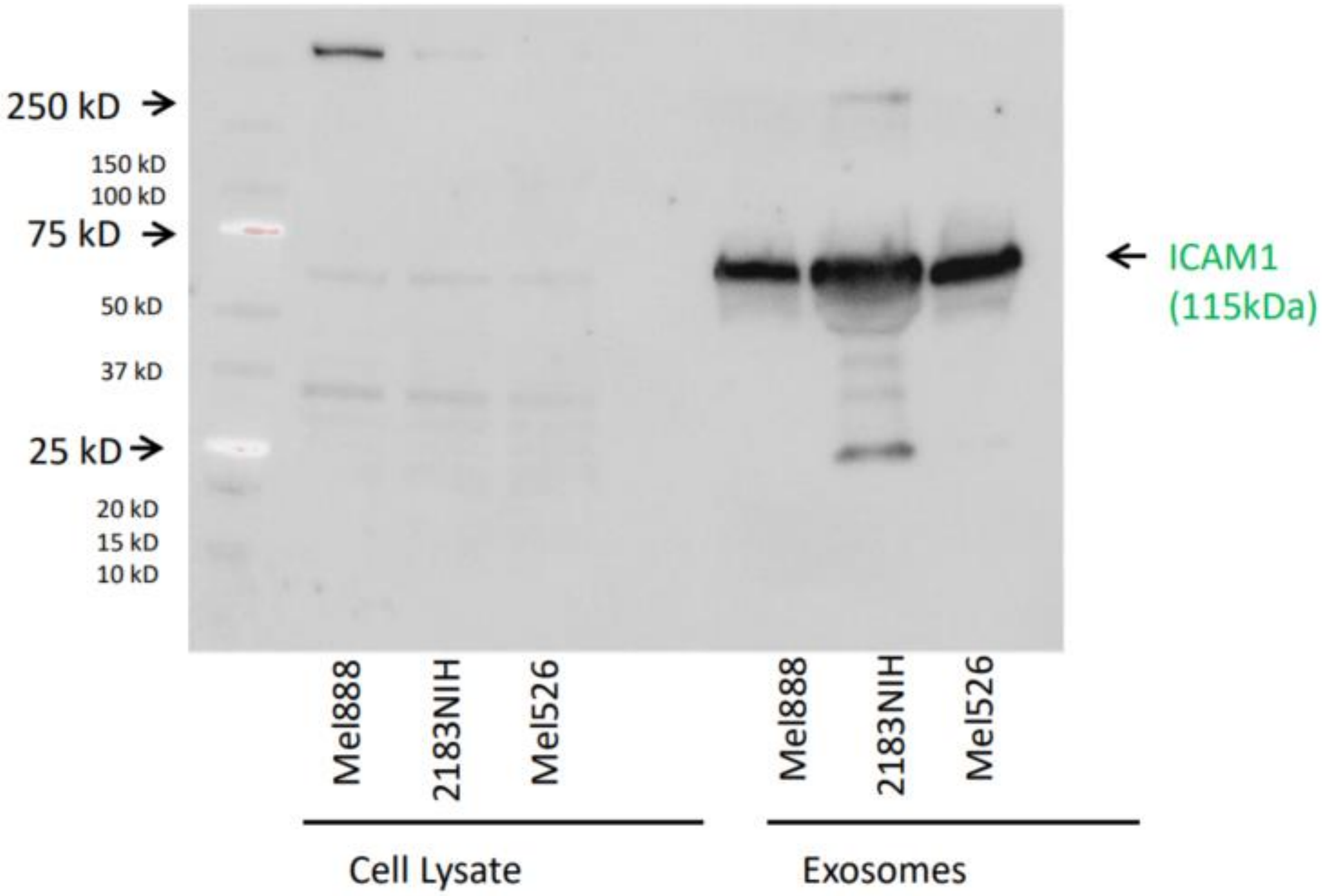
250 kD
150 kD
100 kD
75 kD
50 kD
37 kD
25 kD
20 kD
15 kD
10 kD
ICAM1 (115kDa)
Mel888
2183NIH
Mel526
Mel888
2183NIH
Mel526
Cell Lysate
Exosomes

Figure 2 C, uncropped

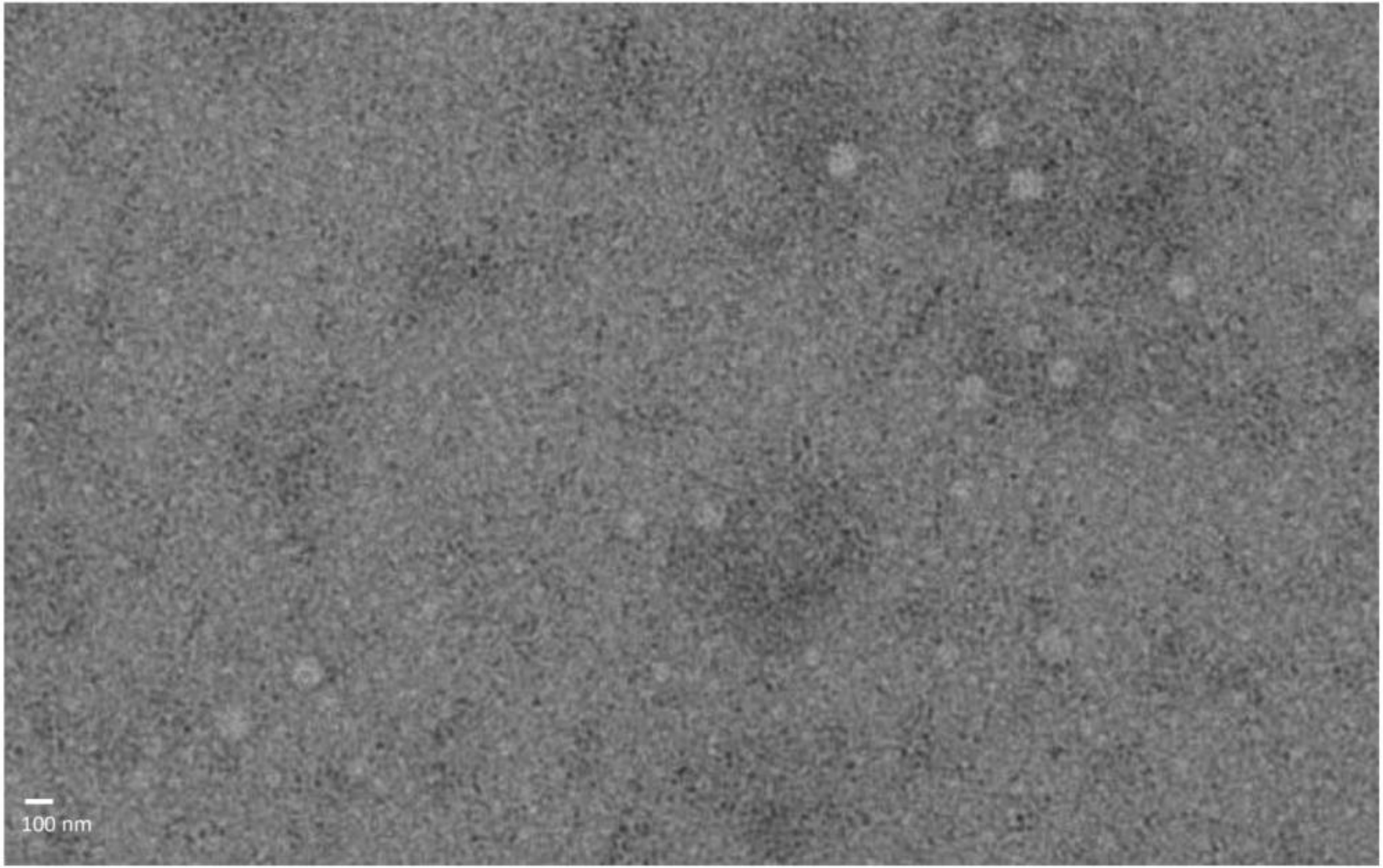

Supplement: Supplementary file 1 [file cancers-17-02509-s001.zip › cancers-3737026 Supplementary Materials/Figure S2.pdf]
