# Supplementary material for: Scaffold-Free Functional Deconvolution Identifies Clinically Relevant Metastatic Melanoma EV Biomarkers
Source: Cancers (Basel). 2025 Jul 30;17(15):2509. doi: 10.3390/cancers17152509 (PMC12345765; doi:10.3390/cancers17152509)
Supplement: Supplementary file 1 [file cancers-17-02509-s001.zip › cancers-3737026 Supplementary Materials/Table S1.pdf]

Table S1. Patient demographics of TCGA melanoma cohort

Age, median, (range)	65 (24–90)
Sex, n, (%)	
Male	61 (59.2)
Female	42 (40.8)
Race, n, (%)	
White	94 (91.3)
Asian	7 (6.8)
Unknown	2 (1.9)
Clark level, n, (%)	
II	1 (1.0)
III	14 (13.6)
IV	39 (37.9)
V	21 (20.4)
Unknown	28 (27.2)
Tumor site, n, (%)	
Extremities	41 (39.8)
Head and Neck	8 (7.8)
Trunk	47 (45.6)
Other	5 (4.9)
Unknown	2 (1.9)
Tumor ulceration, n, (%)	
Yes	76 (73.8)
No	13 (12.6)
Unknown	14 (13.6)
pT, n, (%)	
T1	1 (1.0)
T2	5 (4.9)
T3	10 (9.7)
T4	85 (82.5)
Unknown	2 (1.9)
pN, n, (%)	
N0	58 (56.3)
N1	8 (7.8)
N2	10 (9.7)
N3	11 (10.7)
NX or unknown	16 (15.5)
pM, n, (%)	
M0	98 (95.1)
M1a	1 (1.0)
M1b	1 (1.0)
M1c	1 (1.0)
Unknown	2 (1.9)
pAJCC Stage, n, (%)	
Stage I	2 (1.9)
Stage II	66 (64.1)
Stage III	27 (26.2)
Stage IV	3 (2.9)
Unknown	5 (4.9)
