# Supplementary figures and images for: Scaffold-Free Functional Deconvolution Identifies Clinically Relevant Metastatic Melanoma EV Biomarkers
Source: Cancers (Basel). 2025 Jul 30;17(15):2509. doi: 10.3390/cancers17152509 (PMC12345765; doi:10.3390/cancers17152509)

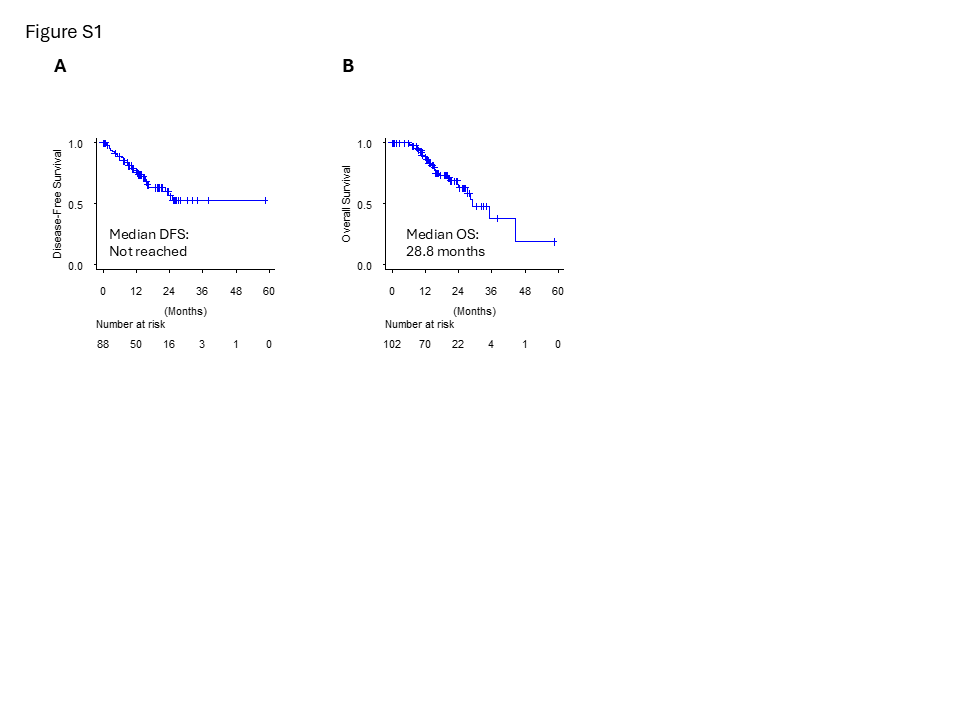

Supplement: Supplementary file 1 [file cancers-17-02509-s001.zip › cancers-3737026 Supplementary Materials/Figure S1.TIF]
